# Early restoration of parvalbumin interneuron activity prevents memory loss and network hyperexcitability in a mouse model of Alzheimer’s disease

**DOI:** 10.1038/s41380-019-0483-4

**Published:** 2019-08-20

**Authors:** Sara Hijazi, Tim S. Heistek, Philip Scheltens, Ulf Neumann, Derya R. Shimshek, Huibert D. Mansvelder, August B. Smit, Ronald E. van Kesteren

**Affiliations:** 1 Department of Molecular and Cellular Neurobiology, Center for Neurogenomics and Cognitive Research, Amsterdam Neuroscience, VU University Amsterdam, Amsterdam, the Netherlands; 2 Department of Integrative Neurophysiology, Center for Neurogenomics and Cognitive Research, Amsterdam Neuroscience, VU University Amsterdam, Amsterdam, the Netherlands; 3 Alzheimer Center and Department of Neurology, Amsterdam Neuroscience, VU University Medical Center, Amsterdam, the Netherlands; 4 Neuroscience Research, Novartis Institutes for BioMedical Research, Basel, Switzerland

**Keywords:** Neuroscience, Physiology

## Abstract

Neuronal network dysfunction is increasingly recognized as an early symptom in Alzheimer’s disease (AD) and may provide new entry points for diagnosis and intervention. Here, we show that amyloid-beta-induced hyperexcitability of hippocampal inhibitory parvalbumin (PV) interneurons importantly contributes to neuronal network dysfunction and memory impairment in APP/PS1 mice, a mouse model of increased amyloidosis. We demonstrate that hippocampal PV interneurons become hyperexcitable at ~16 weeks of age, when no changes are observed yet in the intrinsic properties of pyramidal cells. This hyperexcitable state of PV interneurons coincides with increased inhibitory transmission onto hippocampal pyramidal neurons and deficits in spatial learning and memory. We show that treatment aimed at preventing PV interneurons from becoming hyperexcitable is sufficient to restore PV interneuron properties to wild-type levels, reduce inhibitory input onto pyramidal cells, and rescue memory deficits in APP/PS1 mice. Importantly, we demonstrate that early intervention aimed at restoring PV interneuron activity has long-term beneficial effects on memory and hippocampal network activity, and reduces amyloid plaque deposition, a hallmark of AD pathology. Taken together, these findings suggest that early treatment of PV interneuron hyperactivity might be clinically relevant in preventing memory decline and delaying AD progression.

## Introduction

Alzheimer’s disease (AD) accounts for the most common form of dementia with a population prevalence that increases rapidly. Recent clinical studies have described early prodromal AD stages that precede dementia, creating new opportunities for the development of novel treatment, and prevention strategies [1, 2]. Interestingly, early prodromal symptoms include alterations in neuronal network activity, as observed both in AD patients and in animal models of AD, and these alterations may be responsible for early cognitive impairment [3–7]. As such, neuronal network dysfunction might be a risk factor for AD, a biomarker for identifying early stage AD or people at risk of developing AD, and a novel therapeutic entry point for effective treatment of AD [8–11].

Neuronal network alterations in AD have been identified as impairments in both excitatory and inhibitory synaptic transmission [4, 12]. Typically, studies have focused on aberrant increases in excitatory neuronal activity, exploring the hypothesis that enhanced glutamatergic transmission is driving AD pathogenesis [12–14]. Indeed, epileptiform discharges and seizures are highly prevalent in AD patients [11, 15–17] and the incidence of unprovoked seizures is more than fivefold larger in late-onset sporadic AD patients compared with age-matched controls [11, 17, 18]. Likewise, animal models of AD, in particular models with increased amyloid-beta (Aβ) deposition, suffer from increased epileptic susceptibility and seizure incidence [12, 19, 20]. Therefore, reducing glutamatergic transmission and controlling epileptic activity have been extensively tested as potential treatment strategies [15, 21–23]. While alterations in inhibitory activity were also observed, they were initially suggested to reflect compensatory changes in response to glutamatergic dysfunction [12]. Recently, however, inhibitory network dysfunction has been emphasized as a potential causal event in early AD pathogenesis [24–30].

Parvalbumin (PV) neurons are abundant GABAergic inhibitory interneurons that deliver feedback and feedforward inhibition to excitatory pyramidal neurons in many brain areas, including the hippocampus [31, 32]. As such, they play crucial roles in determining oscillatory network activity and regulating plasticity following behavioral learning [33–36]. PV interneuron dysfunction has previously been associated with AD [10, 27, 28, 37, 38]; however, it is still not clear how PV interneuron function is altered in early AD, or how these alterations contribute to AD progression. We here demonstrate that Aβ-induced hyperexcitability of hippocampal PV interneurons plays a causal role in the early cognitive deficits observed in APP/PS1 mice. We show that restoring PV interneuron function using chemogenetics rescues spatial memory impairments in APP/PS1 mice. Our data furthermore indicate that early reinstatement of PV interneuron activity has long-term beneficial effects on hippocampal memory and network function, reduces soluble Aβ levels, and delays the progression of amyloid plaque pathology. Early hyperexcitable PV interneurons thus represent an interesting target for early AD detection and treatment.

## Results

### APP/PS1 mice show hippocampal PV interneuron hyperexcitability at 15–17 weeks of age

APP/PS1 transgenic mice were crossed with PV-Cre transgenic mice, generating an APP/PS1-PV-Cre mouse model that allows for the specific detection and genetic manipulation of PV interneurons in a progressive amyloidosis background. Soluble Aβ levels in the hippocampus of APP/PS1-PV-Cre mice at 16 weeks of age were found similar to those measured in APP/PS1 mice of the same age (Supplementary Fig. 1a, b). Also, aggregation of insoluble amyloid plaques in the hippocampus was detected at 24 weeks and not yet at 16 weeks of age, similar as observed in APP/PS1 mice (Supplementary Fig. 1a, c, d). To confirm the specific targeting of PV interneurons in this model, the hippocampal CA1 region was bilaterally injected with AAV5-expressing mCherry (hSyn-DIO-mCherry) (Supplementary Fig. 2a–c). More than 84% of PV-expressing interneurons in the CA1 region showed mCherry expression (Supplementary Fig. 2d) and all mCherry-expressing cells recorded showed the typical fast-spiking properties of PV interneurons (Supplementary Fig. 2e–h, Supplementary Table 1), confirming correct targeting of the desired cell type.

**Fig. 1:**
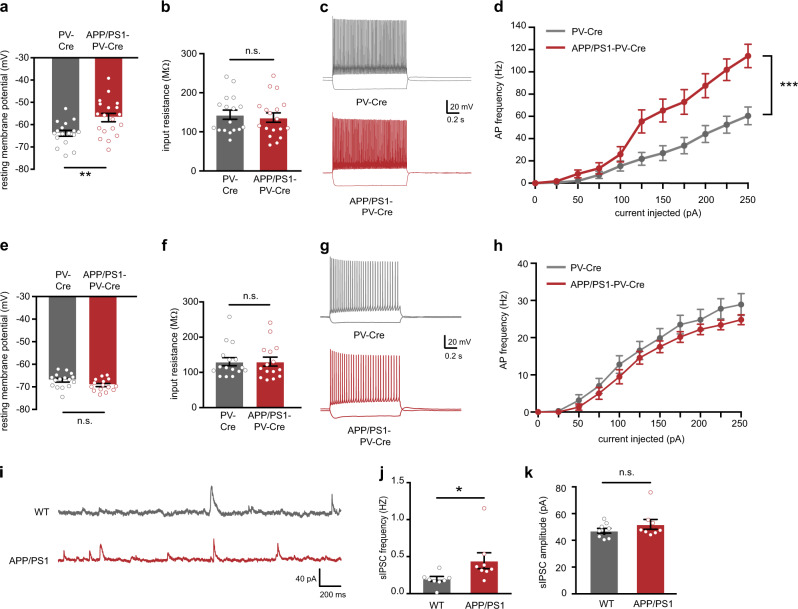
Increased hippocampal PV interneuron excitability and altered hippocampal inhibitory activity in APP/PS1 at 15–17 weeks of age. **a** PV interneuron resting membrane potential was significantly increased in APP/PS1-PV-Cre mice at 15–17 weeks of age compared with WT-PV-Cre controls (Student’s *t* test: *n* = 17/19 cells from four mice per genotype, **p < 0.01). **b** There was no difference in PV interneuron input resistance between APP/PS1-PV-Cre mice and WT-PV-Cre controls (Student’s *t* test: *n* = 17/19 cells from four mice per genotype, *p* = 0.668). **c** Voltage responses to 1 s hyperpolarizing or depolarizing current steps from a PV interneuron in a WT-PV-Cre mouse (gray) or an APP/PS1-PV-Cre mouse (red). **d** Average AP frequency in response to 0–250 pA depolarizing current steps illustrating a significant increase in PV interneuron excitability in APP/PS1-PV-Cre mice compared with WT-PV-Cre controls (*genotype* *×* *current* two-way repeated measures ANOVA: *n* = 17/19 cells from four mice per genotype, *F*_10,180_ = 8.03, ***p < 0.001). **e**, **f** Pyramidal neuron resting membrane potential **e** and input resistance **f** were unaltered in APP/PS1-PV-Cre mice at 15–17 weeks of age compared with WT-PV-Cre controls (Student’s *t* test: *n* = 16/15 cells from four mice per genotype, *p* = 0.084 and *p* = 0.992). **g** Voltage responses to 1 s hyperpolarizing or depolarizing current steps from a pyramidal neuron in a WT-PV-Cre mouse (gray) or an APP/PS1-PV-Cre mouse (red). **h** AP frequency in response to 0–250 pA depolarizing current steps illustrating no significant change in pyramidal neuron excitability in APP/PS1-PV-Cre mice compared with WT-PV-Cre controls (*genotype* *×* *current* two-way repeated measures ANOVA: *n* = 16/15 cells from four mice per genotype, *F*_10,160_ = 0.814, *p* = 0. 615). **i** Example traces of spontaneous inhibitory postsynaptic currents (sIPSC) recorded from hippocampal pyramidal neurons in APP/PS1 mice (red) and WT controls (gray) at 15–17 weeks of age. **j** Increased sIPSC frequency in APP/PS1 mice compared with WT controls (Mann–Whitney test: *n* = 7/8 cells from three mice per genotype, *p < 0.05). **k** No alterations were observed in the amplitudes of both sIPSCs (Mann–Whitney test: *n* = 7/8 cells from three mice per genotype, *p* = 0.370)

We then expressed mCherry in hippocampal PV neurons in APP/PS1-PV-Cre mice and PV-Cre control mice and subsequently performed whole-cell patch-clamp recordings. We observed an early increase in the excitability of PV interneurons at 15–17 weeks of age compared with WT-PV-Cre controls (Supplementary Table 2). In particular, PV interneurons of APP/PS1-PV-Cre mice had a significantly depolarized resting membrane potential (Fig. 1a) with unaltered input resistance (Fig. 1b), a significantly larger increase in action potential firing frequency with increasing current injections (Fig. 1c, d), and a significantly smaller action potential half-width (Supplementary Table 2), indicating a hyperexcitable PV interneuron phenotype. No changes in the excitability of pyramidal neurons was observed at this age (Fig. 1e–h, Supplementary Table 2), and neither PV interneurons nor pyramidal cells were affected at 7–9 weeks of age (Supplementary Fig. 3, Supplementary Table 3). We also quantified PV immunofluorescence levels in hippocampal sections of 15–17-week-old APP/PS1 and APP/PS1-PV-Cre mice. PV immunofluorescence has been demonstrated to correlate well with GABA synthesis and reflect hippocampal PV interneuron activity [33]. Anti-PV staining in the hippocampal CA1 area was overall significantly increased in both mouse lines compared with their respective WT controls, while no changes were detected in total number of PV neurons (Supplementary Fig. 4), suggesting that in 15–17-week-old APP/PS1 mice hippocampal PV interneurons are indeed also more active in vivo.

To investigate whether the observed increase in PV interneuron excitability would affect inhibitory transmission in the hippocampus, we recorded spontaneous inhibitory postsynaptic currents (sIPSCs) from hippocampal pyramidal neurons. Consistent with increased PV interneuron activity, we observed a significant increase in the frequency of sIPSCs received by CA1 pyramidal neurons of 15–17-week-old APP/PS1 mice compared with WT controls (Fig. 1i, j). sIPSC amplitudes were not affected (Fig. 1k), suggesting an increase in either the number of inhibitory synaptic inputs or the firing frequency of presynaptic inhibitory interneurons. We found no differences in the frequency or amplitudes of spontaneous excitatory postsynaptic currents (sEPSCs) recorded at this age (Supplementary Fig. 5). Taken together, our findings demonstrate that hippocampal PV interneurons in APP/PS1 mice become hyperexcitable at 15–17 weeks of age and increase inhibitory transmission in the hippocampus.

### Early hippocampal PV interneuron hyperexcitability in APP/PS1 mice depends on soluble Aβ

**Fig. 2:**
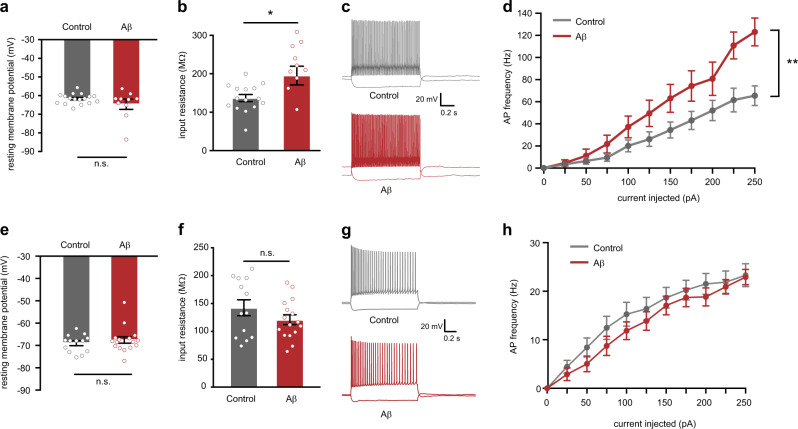
Exogenously applied Aβ increases PV interneuron but not pyramidal neuron excitability in hippocampal slices of wild-type mice. Passive and active membrane properties were measured 1 h after Aβ application. **a** PV interneuron resting membrane potential was not affected by Aβ (Student’s *t* test: *n* = 17/10 cells from 4/3 mice per group, *p* *=* 0.180) but membrane input resistance (**b**) was significantly increased in PV interneurons that were incubated in 1 μM Aβ peptides compared with cells that were incubated with control peptides (Student’s *t* test: *n* = 17/10 cells from 4/3 mice per group, *p < 0.05). **c** Voltage responses to 1 s hyperpolarizing or depolarizing current steps from a PV interneuron recorded from slices incubated with control peptides (gray) or with 1 μM Aβ (red). **d** AP frequency in response to 0–250 pA depolarizing current steps showing a significant increase in PV interneuron excitability in slices incubated in 1 μM Aβ compared with control slices *(genotype* *×* *current* two-way repeated measures ANOVA: *n* = 17/10 cells from 4/3 mice per group, *F*_10,250_ = 4.50, *p* = 0.006). **e** Application of 1 μM Aβ peptides did not alter resting membrane potential and (**f**) did not affect membrane input resistance in pyramidal neurons (Student’s *t* test: *n* = 13/16 cells from four mice per group; *p* = 0.449 and *p* = 0.195). **g** Voltage responses to 1 s hyperpolarizing or depolarizing current steps from a pyramidal neuron recorded from slices incubated with control peptides (gray) or with 1 μM Aβ (red). **h** AP frequency in response to 0–250 pA depolarizing current steps showing no significant change in pyramidal neuron excitability in slices incubated in 1 μM Aβ compared with control slices (*genotype* *×* *current* two-way repeated measures ANOVA: *n* = 13/16 cells from four mice per group, *F*_10,250_ = 4.98, *p* = 0.494)

**Fig. 3:**
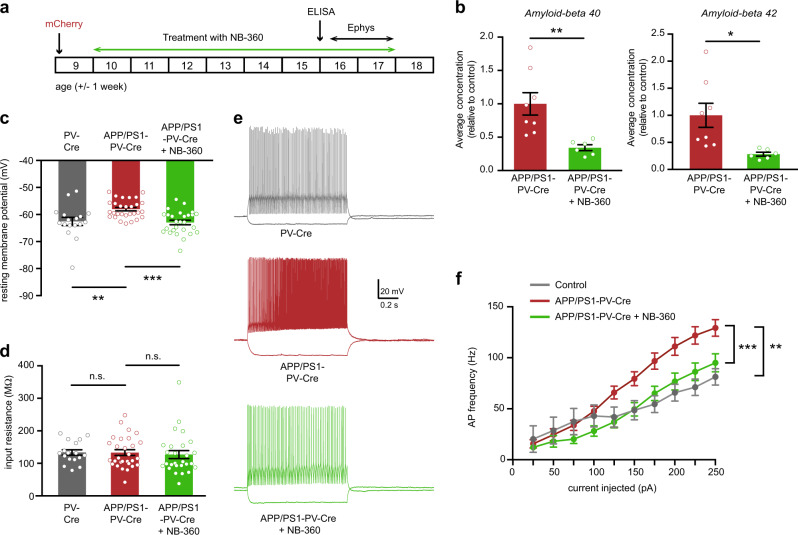
Reducing Aβ levels restores PV neuron excitability in APP/PS1 mice in vivo. **a** Animals were injected with mCherry-expressing virus at 8–10 weeks of age. Mice were then treated with food pellets containing BACE1 inhibitor NB-360 or control food pellets. Mice were sacrificed at 16 weeks of age, after 6 weeks of treatment, for ELISA measurements or at 15–17 weeks of age, after 6–8 weeks of treatment, for electrophysiological recordings. **b** Levels of Aβ_1–40_ (left panel) and Aβ_1–42_ (right panel) in the hippocampus of 16-week-old APP/PS1-PV-Cre that received NB-360 treatment were significantly decreased compared with APP/PS1-PV-Cre mice that received vehicle treatment (Student’s *t* test; *n* = 8/6 mice per group, *p < 0.05; **p < 0.01). **c** PV interneuron resting membrane potential was restored to wild-type levels in NB-360-treated APP/PS1-PV-Cre mice (two-way ANOVA: *n* = 15/27/28 cells from 3/5/5 mice per group, *F*_2,70_ = 9.61, *p* = 0.0002; post-hoc LSD test: **p < 0.01; ***p < 0.001). **d** There was no difference in PV interneuron input resistance between groups (two-way ANOVA: *n* = 15/27/28 cells from 3/5/5 mice per group, *F*_2,70_ = 0.20, *p* = 0.8203). **e** Voltage responses to 1 s hyperpolarizing or depolarizing current steps from a PV interneuron in wild-type PV-Cre (gray), vehicle-treated APP/PS1-PV-Cre (red), and NB-360-treated APP/PS1-PV-Cre (green) mice. **f** Average action potential (AP) frequency in response to 0–250 pA depolarizing current steps illustrating a significant rescue of PV interneuron excitability in NB-360-treated APP/PS1-PV-Cre mice compared with vehicle-treated APP/PS1-PV-Cre mice (*genotype* *×* *current* two-way repeated measures ANOVA: 15/27/28 cells from 3/5/5 mice per group, *F*_2,68_ = 8.48, *p* = 0.001; post-hoc LSD test: **p < 0.01; ***p < 0.001)

To test whether increased PV neuron hyperexcitability could be a direct consequence of high Aβ levels, we exposed hippocampal slices of WT-PV-Cre mice to a mixture of Aβ_1–40_ and Aβ_1–42_ peptides at 1 μM and performed whole-cell patch-clamp recordings. mCherry-positive PV interneurons that were exposed to Aβ for 1 h showed increased excitability compared with PV cells exposed to a control peptide (Supplementary Table 4). Specifically, Aβ increased the input resistance of PV interneurons (Fig. 2b) without affecting the resting membrane potential, contrary to what was observed in APP/PS1-PV-Cre slices (Fig. 2a). However, due to larger input resistance after exposure to Aβ, less current was required to elicit the same extent of depolarization, resulting in a marked increase in action potential firing with increasing current injections compared with control neurons, as observed in APP/PS1-PV-Cre slices (Fig. 2c, d). In contrast, no alterations in input resistance, resting membrane potential or action potential firing were observed in pyramidal neurons due to Aβ exposure (Fig. 2e–h, Supplementary Table 4). To test whether increased Aβ levels also contribute to PV neuron hyperexcitability in vivo, APP/PS1-PV-Cre mice were treated for 6 weeks with NB-360, a potent BACE1 inhibitor that reduces Aβ levels and amyloid pathology and rescues neuronal network and behavioral deficits in mouse models of AD [39–42]. Mice were sacrificed directly following the treatment, at 15–17 weeks of age (Fig. 3a). ELISA measurements confirmed a significant reduction in soluble hippocampal Aβ_1–40_ and Aβ_1–42_ levels by 65.7% and 71.7%, respectively (Fig. 3b, Supplementary Table 5). Patch-clamp recordings from control APP/PS1-PV-Cre mice confirmed the early hyperexcitable phenotype of hippocampal PV neurons. PV neurons showed a depolarized resting membrane potential, unaltered input resistance and increased action potential firing frequency with increasing current injections (Fig. 3c–f, Supplementary Table 6). NB-360 treatment completely restored PV neuron properties to wild-type levels (Fig. 3c–f, Supplementary Table 6). Together, these data show that early increased soluble Aβ levels in APP/PS1 mice may directly contribute to PV interneuron hyperexcitability in vivo.

### APP/PS1 mice show spatial memory impairments at 15–17 weeks of age

**Fig. 4:**
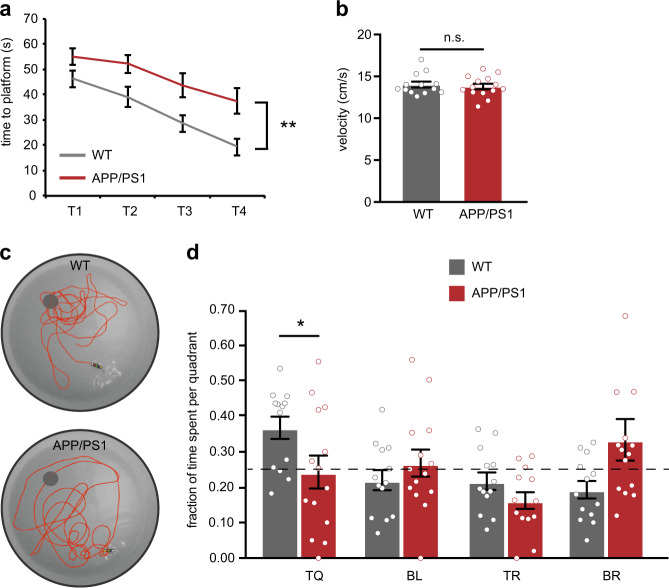
Impaired hippocampal spatial learning and memory in APP/PS1 at 15–17 weeks of age. **a** Spatial learning was assessed at 15–17 weeks of age in a Morris water maze (MWM) by measuring the time required to find the hidden platform on 4 consecutive training days (T1–4). APP/PS1 mice showed a significant learning impairment (*genotype* *×* *training* two-way repeated measures ANOVA: *n* = 13/14 mice per genotype, *F*_1,27_ = 14.45, *p* = 0.001). **b** Average swim velocity during the training did not differ between APP/PS1 mice and WT controls (Student’s *t* test: *n* = 13/14 mice per genotype, *p* = 0.63). **c** Spatial memory was assessed measuring the relative time spent in each quadrant of the maze during a 1-min probe trial with the platform removed. **d** WT mice spent significantly more time in the target quadrant (TQ) compared with APP/PS1 mice (BR, bottom-right; BL, bottom-left; TR, top-right) (Student’s *t* test: *n* = 13/14 mice per genotype, *p < 0.05). Compared with chance level (dashed line), WT mice spent significantly more time in the target quadrant and APP/PS1 mice did not (Student’s *t* test: *p* < 0.05)

We next investigated how early increased excitability of hippocampal PV interneurons in APP/PS1 mice correlates with memory impairment. In a Morris water maze (MWM) test, APP/PS1 mice at 15–17 weeks of age required more time to find the hidden platform during training (Fig. 4a, Supplementary Table 7). No differences were observed in average swim velocity (Fig. 4b, Supplementary Table 7). In the probe test, APP/PS1 mice performed at chance level, whereas WT mice spent significantly more time in the target quadrant where the platform was originally located (Fig. 4c, d), indicating a memory of the platform location. Thus, PV hyperexcitability in APP/PS1 mice coincides with spatial learning and memory impairments.

### Chemogenetic inhibition of hippocampal PV interneurons rescues spatial learning and memory in APP/PS1 mice

To test whether PV interneuron hyperexcitability is causally involved in the observed memory impairment, we aimed to rescue memory by specifically reducing hippocampal PV interneuron activity in APP/PS1 mice. First, the inhibitory hM4Di receptor was bilaterally expressed in CA1 hippocampal PV interneurons of PV-Cre mice using AAV hSyn-DIO-hM4Di-mCherry (Supplementary Fig. 6a–d). On average, 83% of the PV interneurons in the CA1 area expressed hM4Di-mCherry, and 87% mCherry-positive cells expressed PV (Supplementary Fig. 6e), confirming both the efficacy and the specificity of the approach. In hippocampal slice preparations of these animals, bath application of clozapine-N-oxide (CNO) at 50 μM significantly reduced the resting membrane potential and induced hyperpolarization of mCherry-positive cells (Supplementary Fig. 6f, g), confirming functionality of the hM4Di receptor in PV cells.

**Fig. 5:**
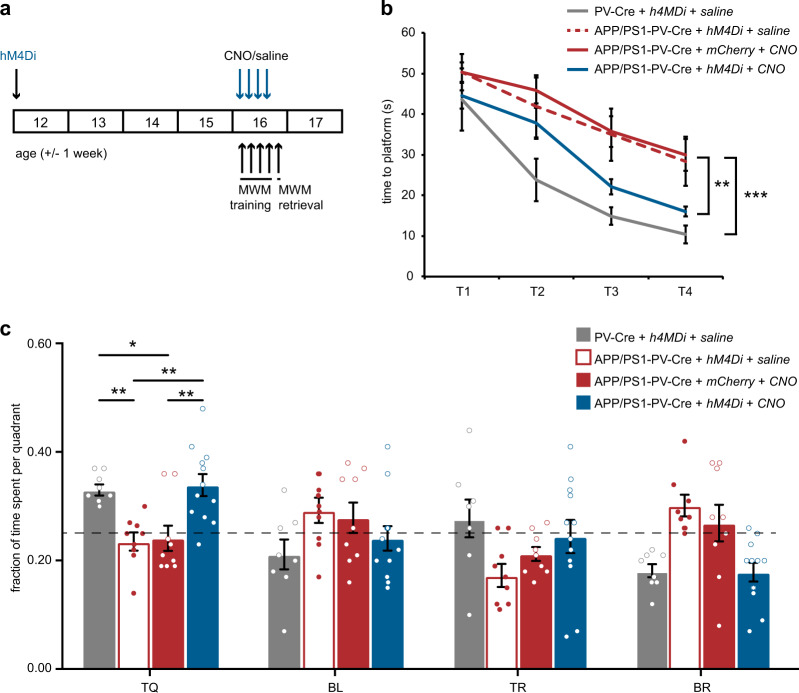
Chemogenetic inhibition of hippocampal PV interneurons during learning rescues spatial memory deficits in APP/PS1 mice. **a** Animals were injected with hM4Di virus at 11–13 weeks of age and tested in an MWM test four weeks later. CNO (5 mg/kg) or saline were i.p. injected 30 min prior to each training session. **b** Spatial learning was assessed measuring the time required to find the hidden platform on 4 consecutive training days (T1–4). APP/PS1-PV-Cre mice that expressed hM4Di and received CNO injections showed a significant learning improvement compared with hM4Di-expressing APP/PS1-PV-Cre mice that received saline or mCherry-expressing mice that received CNO, and were comparable to WT-PV-Cre mice that received saline injections in the last two training sessions (*group* *×* *training* two-way repeated measures ANOVA: *n* = 8/9/9/12 mice per group, *F*_6,66_ = 1.97, *p* = 0.003; post-hoc LSD test: *p < 0.05, **p < 0.01). **c** During the 1-min probe trial, CNO-treated APP/PS1-PV-Cre mice expressing hM4Di spent significantly more time in the target quadrant (TQ) compared with saline-treated APP/PS1-PV-Cre mice expressing hM4Di or CNO-treated APP/PS1-PV-Cre mice expressing mCherry, and performed similar to saline-treated WT-PV-Cre mice (two-way ANOVA: *n* = 8/9/9/12 mice per group, F_3,99_ = 3.79, *p* = 0.013; post-hoc LSD test: *p < 0.05, **p < 0.01). Compared with chance level (dashed line), CNO-treated APP/PS1-PV-Cre expressing hM4Di and saline-treated WT-PV-Cre mice spent significantly more time in the target quadrant, and the two control groups did not (Student’s *t* test: *p* < 0.05 for WT-PV-Cre mice and *p* < 0.01 for hM4Di-expressing APP/PS1-PV-Cre)

We then expressed hM4Di, or the control construct hSyn-DIO-mCherry, in APP/PS1-PV-Cre and WT-PV-Cre mice and performed an MWM test. Thirty minutes prior to each training session, APP/PS1-PV-Cre mice received either CNO (5 mg/kg) or saline injections, whereas WT-PV-Cre mice received saline injections (Fig. 5a). APP/PS1-PV-Cre mice that expressed hM4Di and had received CNO injections prior to training showed significantly faster learning than hM4Di-expressing mice that had received saline or mCherry-expressing mice that had received CNO (Fig. 5b). During the probe test, hM4Di-expressing APP/PS1-PV-Cre mice that had received CNO spent significantly more time in the target quadrant and were behaviorally indistinguishable from WT-PV-Cre mice, whereas both the saline-injected APP/PS1-PV-Cre mice, and the CNO-injected APP/PS1-PV-Cre mice expressing mCherry only, performed at chance level (Fig. 5c). Importantly, inhibiting PV interneurons during training in WT-PV-Cre mice had no effect on learning or memory (Supplementary Fig. 7). These findings demonstrate that decreasing the activity of hyperexcitable PV interneurons during learning rescues spatial learning and memory deficits specifically in APP/PS1 mice.

### Spatial learning and memory, PV interneuron intrinsic properties, and inhibitory synaptic transmission are fully restored by prior chemogenetic inhibition of PV cells

**Fig. 6:**
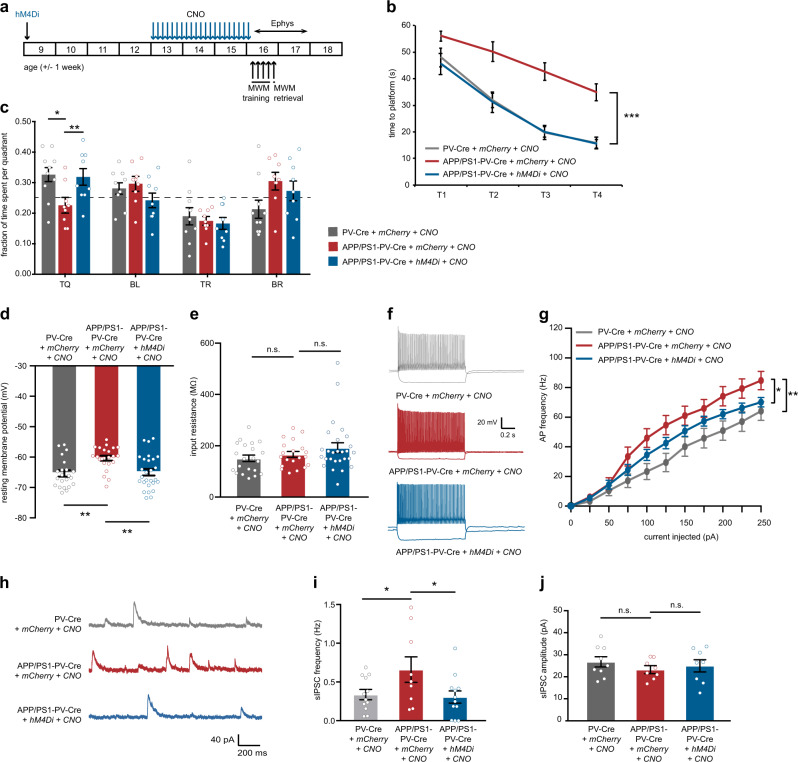
Prolonged chemogenetic inhibition of hippocampal PV interneurons rescues spatial learning and memory and restores PV neuron intrinsic properties and overall network properties. **a** Animals were injected with hM4Di or mCherry-control virus at 8–10 weeks of age. After 4 weeks, CNO (3 mg/kg) or saline were i.p. injected daily for a period of 3 weeks. An MWM test was performed at week 15–17, 2 days after discontinuation of CNO injections. Electrophysiological recordings were performed in a separate cohort of animals at 2–15 days after discontinuation of CNO injections. **b** Spatial learning was assessed measuring the time required to find the hidden platform on 4 consecutive training days (T1–4). APP/PS1-PV-Cre mice that expressed hM4Di and had received CNO injections showed a significant learning improvement compared with mCherry-expressing APP/PS1-PV-Cre mice and were indistinguishable from mCherry-expressing WT-PV-Cre mice (*group* *×* *training* two-way repeated measures ANOVA: *n* = 10/9/9 per group, *F*_6,72_ = 1.48, *p* = 0.000; post-hoc LSD test: ***p < 0.001). **c** During the 1-min probe trial, CNO-treated APP/PS1-PV-Cre mice expressing hM4Di spent significantly more time in the target quadrant (TQ) compared with APP/PS1-PV-Cre mice expressing mCherry, and performed similar to WT-PV-Cre mice expressing mCherry (two-way ANOVA: *n* = 10/9/9 mice per group, F_2,27_ = 6.09, *p* = 0.007; *p*ost-hoc LSD test: *p < 0.05, **p < 0.01). Compared with chance level (dashed line), CNO-treated APP/PS1-PV-Cre mice expressing hM4Di and WT-PV-Cre mice expressing mCherry spent significantly more time in the target quadrant, while APP/PS1-PV-Cre mice expressing mCherry did not (Student’s *t* test: *p* < 0.05). **d** PV interneuron resting membrane potential was restored to wild-type levels in CNO-treated APP/PS1-PV-Cre mice expressing hM4Di (two-way ANOVA: *n* = 22/22/27 cells from five mice per group, *F*_2,68_ = 7.24, *p* = 0.001; post-hoc LSD test: **p < 0.01). **e** There was no difference in PV interneuron input resistance between groups (two-way ANOVA: *n* = 22/22/27 cells from five mice per group, *F*_2,65_ = 1.89, *p* = 0.159). **f** Voltage responses to 1 s hyperpolarizing or depolarizing current steps from a PV interneuron in WT-PV-Cre (gray), CNO-injected control APP/PS1-PV-Cre (red), and CNO-injected APP-PS1-PV-Cre (blue) mice. **g** Average action potential (AP) frequency in response to 0–250 pA depolarizing current steps illustrating a significant rescue in PV interneuron excitability in CNO-treated APP/PS1-PV-Cre mice expressing hM4Di compared with APP/PS1-PV-Cre mice expressing mCherry control (*genotype* *×* *current* two-way repeated measures ANOVA: *n* = 22/22/27 cells from five mice per group, *F*_4,163_ = 3.19, *p* = 0.010; post-hoc LSD test: *p < 0.05, **p < 0.01). **h** Example traces of spontaneous inhibitory postsynaptic currents (sIPSC) recorded from hippocampal pyramidal neurons in WT-PV-Cre (gray), CNO-injected control APP/PS1-PV-Cre (red), and CNO-injected APP-PS1-PV-Cre (blue) mice. **i** A significant rescue of inhibitory transmission to wild-type levels was observed in sIPSC frequencies in CNO-treated APP/PS1-PV-Cre mice expressing hM4Di compared with APP/PS1-PV-Cre mice expressing mCherry control (two-way ANOVA: *n* = 12/9/12 cells from 5/4/5 mice per group, *F*_2,30_ = 3.37, *p* = 0.047; post-hoc LSD test: *p < 0.05). **j** No significant changes were noted in sIPSC amplitude between groups (two-way ANOVA: *n* = 12/9/12 cells from 5/4/5 mice per group, *F*_2,21_ = 0.540, *p* = 0.590)

We next tested whether prolonged chemogenetic inhibition of hippocampal PV interneurons is sufficient to rescue spatial learning and memory in the subsequent period, i.e., in the absence of acute effects of CNO. APP/PS1-PV-Cre mice expressing hM4Di or mCherry-control virus and WT-PV-Cre mice expressing mCherry-control virus in hippocampal PV interneurons received daily injections of CNO (3 mg/kg) for a period of 3 weeks and were subjected to an MWM test 48 h after the last injections (Fig. 6a). Because of the short half-life of CNO [43], no direct effects can be measured anymore at this moment in time. Similar to acute CNO treatment, APP/PS1-PV-Cre mice that expressed hM4Di and had received CNO injections showed significantly faster learning than mCherry-expressing mice and were indistinguishable from WT-PV-Cre control mice (Fig. 6b). During the probe test, hM4Di-expressing APP/PS1-PV-Cre mice spent significantly more time in the target quadrant, similar to WT-PV-Cre mice, whereas mCherry-expressing APP/PS1-PV-Cre mice performed at chance level (Fig. 6c). A rotarod test was used to confirm normal locomotor behavior in all treatment groups (Supplementary Fig. 8).

Next, we also investigated the intrinsic and network properties of PV interneurons after treatment using whole-cell patch-clamp recording (Fig. 6d–g). Intrinsic properties of PV interneurons were fully restored, showing no difference between CNO-treated APP/PS1-PV-Cre mice and WT-PV-Cre mice, whereas PV interneurons of APP/PS1-PV-Cre mice that received the control treatment showed again a significant increase in excitability compared with both other groups (Supplementary Table 8). Specifically, CNO treatment in hM4Di-expressing APP/PS1-PV-Cre mice rescued PV cell resting membrane potential (Fig. 6d), had no effect on input resistance (Fig. 6e), and significantly reduced action potential firing frequency (Fig. 6f, g) and action potential half-width (Supplementary Table 8), all of which were significantly altered in APP/PS1 mice (cf. Fig. 1a–d and Supplementary Table 2). Recorded sIPSCs from pyramidal neurons in the CA1 region of the hippocampus (Fig. 6h) confirmed a decrease to wild-type levels in the frequency of inhibitory inputs onto pyramidal neurons in hM4Di-expressing APP/PS1-PV-Cre mice (Fig. 6i). sIPSC amplitudes were not different between groups (Fig. 6j). Together, our data demonstrate that preventing hyperactivity of PV interneurons in the hippocampus of APP/PS1 mice by chemogenetic inhibition for a period of 3 weeks is sufficient to restore PV cell physiology and network properties and rescue spatial memory deficits.

### Early restoration of PV interneuron function has long-lasting beneficial effects on learning and memory, hippocampal synaptic transmission and amyloid pathology

**Fig. 7:**
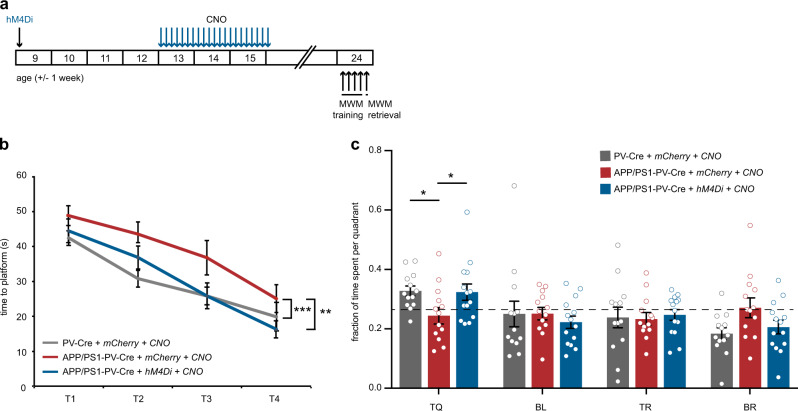
Prolonged inhibition of PV interneurons has long-term beneficial effects on spatial learning and memory. **a** Animals were injected with hM4Di or mCherry-control virus at 8–10 weeks of age. After 4 weeks, CNO (3 mg/kg) or saline was i.p. injected daily for a period of 3 weeks. An MWM test was performed at week 23–25, 8 weeks after discontinuation of CNO injections. **b** Spatial learning was assessed measuring the time required to find the hidden platform on 4 consecutive training days (T1–4). APP/PS1-PV-Cre mice that expressed hM4Di and had received CNO injections showed a significant learning improvement compared with mCherry-expressing APP/PS1-PV-Cre mice and were indistinguishable from mCherry-expressing WT-PV-Cre mice (*group* *×* *training* two-way repeated measures ANOVA: *n* = 13/13/12 mice per group, *F*_6,111_ = 9.58, *p* = 0.000; post-hoc LSD test: *p < 0.05, **p < 0.01). **c** During the 1-min probe trial, CNO-treated APP/PS1-PV-Cre mice expressing hM4Di spent significantly more time in the target quadrant (TQ) compared with APP/PS1-PV-Cre mice expressing mCherry, and performed similar to WT-PV-Cre mice expressing mCherry (two-way ANOVA: *n* *=* 13/13/12 mice per group, *F*_3,108_ = 3.63, *p* = 0.015; post-hoc LSD test: *p < 0.05). Compared with chance level (dashed line), CNO-treated APP/PS1-PV-Cre expressing hM4Di mice and WT-PV-Cre expressing mCherry mice spent significantly more time in the target quadrant, while APP/PS1-PV-Cre expressing mCherry mice did not (Student’s *t* test: *p* < 0.05)

We also tested whether early treatment aimed at restoring hippocampal PV interneuron activity has long-lasting effects in APP/PS1 mice. APP/PS1-PV-Cre mice expressing hM4Di or mCherry-control virus and WT-PV-Cre mice expressing mCherry-control virus received daily injections of CNO for 3 weeks. Following the end of the treatment, mice were left for 8 weeks without any intervention. At weeks 23–25, they were subjected to an MWM test (Fig. 7a). APP/PS1-PV-Cre mice expressing mCherry only showed clear learning deficits, but APP/PS1-PV-Cre mice that expressed hM4Di had no learning impairments and were indistinguishable from WT-PV-Cre mice (Fig. 7b). The probe test confirmed that hM4Di-expressing APP/PS1-PV-Cre mice had also no memory impairments as they spent significantly more time in the target quadrant, similar to WT-PV-Cre mice, whereas mCherry-expressing APP/PS1-PV-Cre mice showed a clear memory deficit and performed at chance level (Fig. 7c).

**Fig. 8:**
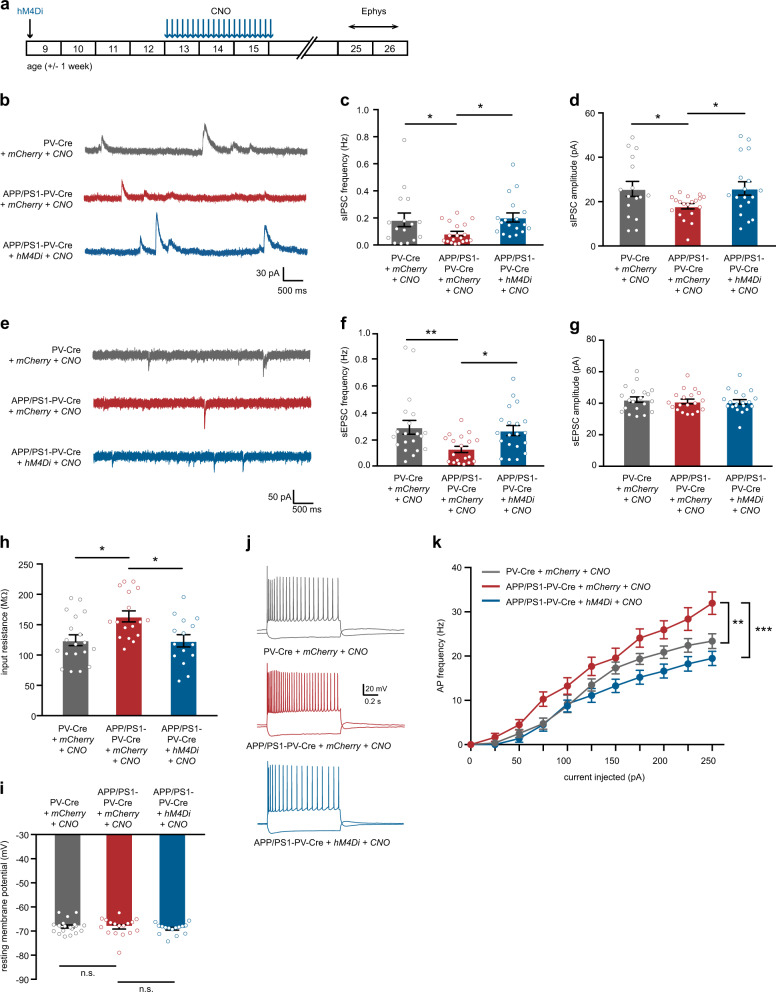
Prolonged inhibition of PV interneurons precludes hippocampal network hyperexcitability. **a** Animals were injected with hM4Di or mCherry-control virus at 8–10 weeks of age. After 4 weeks, CNO (3 mg/kg) or saline was i.p. injected daily for a period of 3 weeks. Electrophysiological recordings were performed at week 24–26, 9–10 weeks after discontinuation of CNO injections. **b**–**d** A significant rescue of inhibitory transmission to WT levels was observed in sIPSC frequencies (**c**) and amplitudes (**d**) in CNO-treated APP/PS1-PV-Cre mice expressing hM4Di compared with APP/PS1-PV-Cre mice expressing mCherry only (frequencies: two-way ANOVA: *n* = 16/18/18 cells from 5/6/5 mice per group, *F*_2,50_ = 3.60, *p* = 0.035; amplitudes: two-way ANOVA: *n* = 16/18/18 cells from 5/6/5 mice per group, *F*_2,50_ = 3.16, *p* = 0.046; post-hoc LSD test: *p < 0.05). **e** Example traces of sEPSCs recorded from hippocampal pyramidal neurons in WT-PV-Cre (gray), CNO-injected control APP/PS1-PV-Cre (red), and CNO-injected APP-PS1-PV-Cre (blue) mice. **f** A significant rescue of excitatory transmission to wild-type levels was observed in sEPSC frequencies in CNO-treated APP/PS1-PV-Cre mice expressing hM4Di compared with APP/PS1-PV-Cre mice expressing mCherry only (two-way ANOVA: *n* = 20/19/20 cells from 5/6/5 mice per group, *F*_2,50_ = 5.00, *p* = 0.010; *p*ost-hoc LSD test: *p < 0.05, **p < 0.01). **g** No significant changes were noted in sEPSC amplitude between groups (two-way ANOVA: *n* = 20/19/20 cells from 5/6/5 mice per group, *F*_2,56_ = 0.244, *p* = 0.784). **h** A significant increase in pyramidal neuron input resistance was observed in APP/PS1-PV-Cre control mice. This increase was rescued by CNO treatment (two-way ANOVA: *n* = 19/17/19 cells from five mice per group, *F*_2,48_ = 6.02, *p* = 0.004; post-hoc LSD test: *p < 0.01). **i** Pyramidal neuron resting membrane potential was unaltered in APP/PS1-PV-Cre mice at 23–25 weeks of age compared with WT-PV-Cre controls (two-way ANOVA: *n* = 19/17/19 cells from five mice per group per group, *F*_2,48_ = 0.413, *p* = 0.663). **j** Voltage responses to 1-s hyperpolarizing or depolarizing current steps from a pyramidal neuron in WT-PV-Cre (gray), CNO-injected control APP/PS1-PV-Cre (red), and CNO-injected APP-PS1-PV-Cre (blue) mice. **k** Average action potential (AP) frequency in response to 0–250 pA depolarizing current steps illustrating a significant rescue in pyramidal neuron excitability in CNO-treated APP/PS1-PV-Cre mice expressing hM4Di compared with APP/PS1-PV-Cre mice expressing mCherry only (*genotype* *×* *current* two-way repeated measures ANOVA: *n* = 19/17/19 cells from five mice per group per group type, *F*_6,151_ = 2.27, *p* = 0.010; post-hoc LSD test: **p < 0.01, ***p < 0.001)

At week 24–27 (9–10 weeks after the treatment had ended), we also recorded inhibitory and excitatory synaptic transmission onto hippocampal CA1 pyramidal cells. We observed a significant decrease in sIPSC frequency and amplitude in mCherry-expressing APP/PS1-PV-Cre (Fig. 8b–d), confirming that at this age, untreated APP/PS1 mice show a clear deficit in inhibitory synaptic transmission [44, 45]. Interestingly, sIPSC frequency was increased to wild-type levels in hM4Di-expressing APP/PS1-PV-Cre mice (Fig. 8b–d), despite the fact that the initial treatment was aimed at inhibiting interneurons, indicating that restoring PV neuron function early has long-term beneficial effects on inhibitory transmission in the hippocampus. sEPSC frequency was also decreased in mCherry-expressing APP/PS1-PV-Cre, and fully restored in hM4Di-expressing APP/PS1-PV-Cre mice (Fig. 8e–g). Previous studies have linked reduced inhibitory synaptic transmission at this stage to impairments in PV neuron activity [27, 38]. We confirmed this by showing that chemogenetic enhancement of PV neuron activity using the hM3Dq receptor in APP/PS1-PV-Cre mice at 23–25 weeks of age rescued learning and memory performance in the MWM test. Thus, prolonged inhibition of PV neurons at week 16 or acute activation of PV neurons at week 24 has similar beneficial effects on hippocampal memory (Supplementary Fig. 9). These results suggest that PV neurons are hypoactive at 24 weeks of age. We then quantified PV immunofluorescence levels in hippocampal sections of 23–25-week-old APP/PS1 mice. Anti-PV staining in the hippocampal CA1 area was overall significantly decreased in APP/PS1 mice compared with WT controls, while no changes were detected in total number of PV neurons (Supplementary Fig. 10), suggesting that in 23–25-week-old APP/PS1 mice hippocampal PV interneurons are indeed hypoactive in vivo.

As many previous studies have highlighted network hyperexcitability in various AD mouse models [12, 19, 46–49], we also measured excitability of pyramidal neurons in the hippocampus at 23–25 weeks of age (Supplementary Table 9). Indeed, we found a significant increase in the input resistance (Fig. 8h) and action potential firing frequency (Fig. 8j, k), but unaltered resting membrane potential (Fig. 8i) of pyramidal neurons in APP/PS1-PV-Cre mice. Importantly, these hyperexcitability measures were all restored to wild-type levels by early CNO treatment targeting hyperexcitable PV neurons at 13–15 weeks of age.

**Fig. 9:**
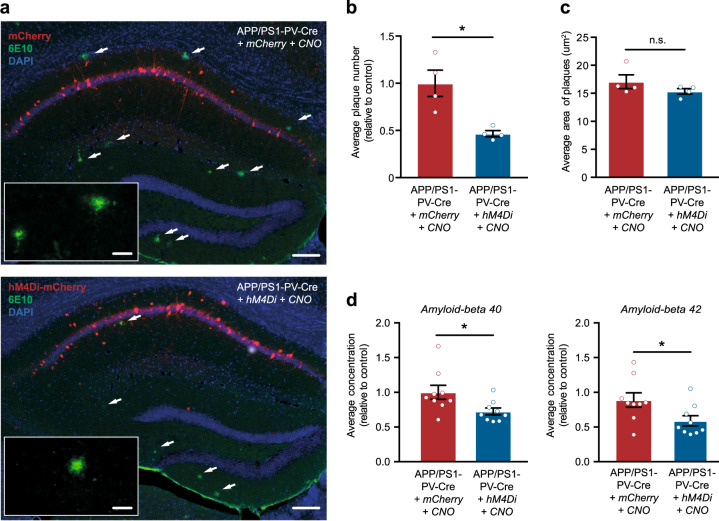
Prolonged inhibition of PV interneurons reduces Aβ levels and amyloid plaques in APP/PS1-PV-Cre mice. **a** Immunohistochemistry with anti-Aβ (6E10, red) antibody in 23–25-week-old mCherry-expressing APP/PS1-PV-Cre mice (upper panel) and hM4Di-expressing APP/PS1-PV-Cre mice (lower panel) 8 weeks following prolonged treatment showing plaques in the hippocampus (white arrows). Scale bar: 100 μm (overview), 20 μm (inset). **b**, **c** APP/PS1-PV-Cre mice expressing hM4Di showed a significant reduction in the average number of Aβ-positive plaques compared with APP/PS1-PV-Cre control mice (**b**), but not in average plaques size (**c**) (Student’s *t* test: *n* = 4 mice per group, *p < 0.05). **d** Relative Aβ_1–40_ (left panel) and Aβ_1–42_ (right panel) levels in the hippocampus of 23–25-week-old APP/PS1-PV-Cre mice 8 weeks following prolonged CNO treatment (Student’s *t* test; *n* = 13 mice per group, *p < 0.05)

Finally, we investigated the long-term effects of restoring PV interneuron activity on the accumulation of soluble and insoluble Aβ in the hippocampus. At the start of the 3-week-treatment period (i.e., at ~16 weeks of age), APP/PS1-PV-Cre mice have no amyloid plaques yet, but at ~24 weeks of age amyloid plaques start to appear in the hippocampus (Supplementary Fig. 1c, d). Interestingly, CNO-treated hM4Di-expresing APP/PS1-PV-Cre mice at 24 weeks of age showed a 52% reduction in plaque numbers in the hippocampus compared with mCherry-expressing APP/PS1-PV-Cre control mice (Fig. 9a, b). Treatment had no effect on plaques size (Fig. 9c). We also found that CNO treatment significantly reduced soluble Aβ_1–40_ levels by 28.6% and soluble Aβ_1–42_ by 41.2% in the hippocampus of hM4Di-expressing APP/PS1-PV-Cre mice compared with mCherry-expressing APP/PS1-PV-Cre control mice, as measured by ELISA (Fig. 9d, Supplementary Table 5). Together, our data demonstrate that restoring PV interneuron activity in the hippocampus of APP/PS1 mice by chemogenetic inhibition for a period of 3 weeks is sufficient to prevent memory decline and pyramidal neuron hyperexcitability in the hippocampus on the long term, and to attenuate the pathological accumulation of Aβ.

## Discussion

### Early and selective hyperactivity of hippocampal PV interneurons in a mouse model of AD

Although the amyloid cascade hypothesis has been leading in AD research over the past 30 years, it is still not clear how Aβ initiates AD-associated neuronal network dysfunction and cognitive impairment. Aberrant inhibitory synaptic transmission is increasingly recognized as an important factor in early AD pathogenesis [12, 24, 29, 45, 46], and PV interneurons in particular have been identified as a potential source of impaired inhibitory transmission in mouse models of AD [10, 27, 28, 38, 50]. Our data confirm that inhibitory transmission in the hippocampus is indeed impaired in APP/PS1 mice at 24 weeks of age and that associated learning and memory impairments can be rescued by chemogenetic activation of hippocampal PV interneurons. However, we provide multiple lines of evidence indicating that PV interneurons are initially hyperexcitable at 15–17 weeks of age. No changes were detected in pyramidal neuron excitability at this age, suggesting that PV interneuron hyperexcitability is an early trigger of hippocampal network alterations in AD. In addition, our data show that the hyperexcitability of PV interneurons in APP/PS1 mice coincides with an increase in spontaneous inhibitory inputs onto hippocampal CA1 pyramidal neurons. No differences in excitatory transmission were detected at this age, indicating again that inhibitory networks are preferentially affected at this young age. Alterations in inhibitory transmission in the hippocampus, in particular by PV interneurons, are linked to spatial memory deficits [24, 27, 51–53]. Indeed, our data show that along with increased PV interneuron excitability and inhibitory activity in hippocampal slices of APP/PS1 mice, learning and memory are impaired. Our PV stainings seem to confirm that PV interneurons are also hyperactive in vivo, but more direct measures of neuronal activity such as calcium imaging using fiber photometry or endomicroscopy in awake and freely moving animals would help validate these findings.

### Early hyperactivity of hippocampal PV interneurons depends on increased levels of Aβ

Previous studies have demonstrated that neurons located in close proximity to amyloid plaques show increased excitability [47, 54]. It has also been shown that exogenous application of Aβ can rapidly induce network hyperactivity and network asynchrony in wild-type mice [24, 54]. Our data show that in APP/PS1 mice at 15–17 weeks of age, which lack amyloid plaque pathology but do show increased brain levels of Aβ and hippocampus-dependent memory impairments, neuronal hyperexcitability is primarily observed in interneurons. Moreover, wild-type hippocampal CA1 PV interneurons, but not pyramidal neurons, responded directly to acute Aβ application with increased excitability. Several biophysical membrane properties of PV interneurons were altered, resulting in a hyperexcitable phenotype with increased action potential firing. Furthermore, reducing Aβ levels in vivo using a BACE1 inhibitor completely restored PV interneuron excitability to wild-type levels, supporting the idea that an aberrant early increase in PV interneuron activity in APP/PS1 mice is linked directly to increased levels of soluble Aβ. Noteworthy, PV interneuron membrane alterations after exogenous Aβ application were not the same as observed in brain slices from APP/PS1 mice. Whereas in APP/PS1 mice increased excitability coincided with a decreased resting membrane potential, direct application of Aβ seemed to enhance excitability by increasing input resistance. This may reflect differences between short-term and long-term cellular adaptations towards Aβ exposure. Further research will be necessary to determine how PV interneurons respond to increased Aβ levels over time, which membrane mechanisms are involved and whether their fast-spiking nonadapting profile, which depends on specific channels [38] and necessitates high-energy turnover [55], could predispose them to Aβ toxicity.

### Reducing PV interneuron activity restores inhibitory transmission and rescues cognitive deficits in APP/PS1 mice

Following these findings, we aimed to test whether hyperexcitable PV interneurons are directly responsible for early cognitive impairment in APP/PS1 mice. Indeed, chemogenetic inhibition of hippocampal PV interneurons during MWM learning rescued learning and memory impairments in APP/PS1-PV-Cre mice at 15–17 weeks of age, confirming that hyperexcitable PV interneurons are causally involved in the observed cognitive deficits at this age. These results confirm previous data showing that hippocampal PV interneuron activity during learning needs to be tightly controlled for functional memory consolidation and retrieval [33]. However, they have limited translational value as the intervention occurred during the memory task. We therefore also tested the effects of prolonged CNO treatment on MWM performance thereafter. Indeed, prolonged inhibition of hippocampal PV interneurons in APP/PS1-PV-Cre mice rescued learning and memory deficits after the treatment. Moreover, it fully restored all physiological properties of hippocampal PV interneurons to wild-type levels, confirming that the treatment was sufficient to prevent the aberrant increase in PV cell excitability. Also, using this intervention, we were able to reinstate inhibitory synaptic transmission in the hippocampus, as reflected by a reduction to wild-type levels of sIPSC frequencies onto pyramidal cells. It would also be interesting to acquire EEG data from these mice to see how increased epileptiform activity correlates with these temporal changes in PV cell activity.

### Prolonged PV interneuron intervention has long-term beneficial effects on learning and memory and hippocampal network properties

We also investigated the long-term effects of chemogenetic PV cell inhibition. Remarkably, APP/PS1 mice that had received such treatment showed no learning or memory impairment at 23–25 weeks of age, i.e., 8 weeks after the end of the treatment. Whereas several studies reported a direct rescue of cognitive deficits in AD mice [24, 27, 29, 56] our results are important in identifying an intervention that also has long-term effects on cognition and may thus be relevant for treatment. Moreover, APP/PS1 mice that received treatment also showed a long-term restoration of hippocampal neuronal function. In particular, the loss of inhibitory synaptic transmission that is observed in many mouse models of AD at this age [24, 27, 45, 57, 58], is prevented by the treatment, and the hyperexcitability of pyramidal neurons, which is also reported in AD mice [3, 12, 19, 47–49, 59], is completely restored. In contrast to the hyperexcitability of pyramidal cells, excitatory transmission in hippocampal slice preparations, as recorded by sEPSC frequency, was decreased in APP/PS1 mice. The decrease, and respective rescue, of both inhibitory and excitatory transmission might thus reflect a compensatory homeostatic network response to pathological alterations in interneuron activity [6, 7]. In conclusion, while several studies have shown that memory loss and network dysfunction can be treated with positive modulators of GABAergic transmission [24, 60, 61], our data indicate that they may actually be prevented when hyperactive PV interneurons are targeted early.

### A biphasic response of hippocampal PV interneurons in AD

We show that activating PV neurons at 24 weeks of age had similar beneficial effects on hippocampal learning and memory as inhibiting PV neurons at 16 weeks of age. These data support biphasic alterations in PV neuron activity in APP/PS1 mice, in which early hyperexcitability of PV interneurons may result in hypoactivity at a later stage, a mechanism that has been proposed for AD previously [45, 50] and reported for other neurodegenerative diseases as well [62]. These findings are also consistent with previous studies showing that increasing GABAergic transmission can restore network function in different mouse models of AD [24, 61, 63], assuming that the hypoactive stage of PV neurons was targeted in these studies. The question remains what initiates the hyperexcitability of PV interneurons and whether other cell types, such as other interneurons or glial cells, contribute to the pathology [29, 64, 65]. In addition, although our data demonstrate that soluble Aβ plays a direct role in PV neuron hyperexcitability, we cannot exclude that increased activity of other inputs, such as excitatory projections from CA3, dentate gyrus, or entorhinal cortex, contribute to early PV neuron hyperactivity in vivo. Indeed, others have demonstrated that excitatory inputs from the entorhinal cortex onto hippocampal PV neurons are affected early in AD mice [28], and it has been suggested that increased inhibitory transmission in the hippocampus is a compensatory response to counteract excitotoxicity [12]. Further studies investigating how PV neurons become dysfunctional over time in vivo would be valuable.

### Prolonged PV interneuron intervention attenuates amyloid pathology

A recent study by Iaccarino et al. [10] demonstrated that optogenetic entrainment of PV interneuron activity in mouse models of AD acutely restores gamma oscillations and also reduces Aβ levels in the hippocampus. Accordingly, we observed a marked decrease in soluble levels of Aβ_1–40_ and Aβ_1–42_ and a significant decrease in the number of amyloid plaques in the hippocampus of APP/PS1 mice in which PV interneurons were inhibited, detectable 8 weeks after the end of the treatment. These results suggest that restoring PV interneuron activity at an early, prepathological stage attenuates the pathological accumulation of Aβ. It remains an open question how exactly PV interneuron activity controls Aβ levels. Iaccarino et al. [10] showed that PV cell-mediated entrainment of gamma oscillations enhances Aβ clearance via activation of microglia. On the other hand, it has also been suggested that hyperactive neurons play a role in amyloid plaque deposition [47]. In particular, hyperactive PV interneurons may directly be responsible for increased Aβ release [66] and hence promote the formation of amyloid plaques. This way, hyperexcitability of PV interneurons and activity-dependent Aβ release may be linked in a detrimental feedforward loop that escapes homeostatic regulation in AD patients.

### Prospective clinical and pharmacological implications

Neuronal hyperexcitability in mouse models of AD is not restricted to interneurons [12, 47, 54], nor are PV interneurons the only interneurons to become dysfunctional [30]. Network remodeling in AD is a complex process involving impaired connectivity at multiple levels [24, 28, 29]. Despite the complexity of neuronal network imbalance in AD, our findings demonstrate that dysfunctional PV interneurons play an early role in this in APP/PS1 mice. Hence, the selective vulnerability of fast-spiking PV interneurons needs to be considered as a potential risk factor in AD, which might contribute to AD symptoms and enhance disease progression. Also, PV interneuron dysfunction, and in particular its consequences for oscillatory network behavior, might be used as an early biomarker for people who are at risk of developing AD.

## Materials and methods

### Animals

APP/PS1 mice (The Jackson Laboratory; strain B6C3-Tg(APPswe,PSEN1dE9)85Dbo/J; stock number 004462) express a chimeric mouse/human APP gene harboring the Swedish double mutation K595N/M596L (APPswe) and a human PS1 gene harboring the exon 9 deletion (PS1dE9), both under the control of the mouse prion protein promoter (MoPrP.Xho) [67–69]. PV-Cre mice (The Jackson Laboratory; strain B6.129P2-Pvalbtm1(cre)Arbr/J; stock number 017320) express Cre recombinase under the control of the mouse parvalbumin (Pvalb) promoter, directing Cre expression specifically to parvalbumin expressing cells [70]. All mouse lines were maintained on a C57BL/6 background. Hemizygous APP/PS1 mice were crossed with hemizygous PV-Cre mice to produce double-transgenic mice and single-transgenic controls. All experiments were performed with single-housed male mice and approved by the Central Committee for Animal Experiments (CCD) and the Animal Welfare Body (IVD) of the VU University Amsterdam.

### Virus

Floxed mCherry (hSyn-DIO-mCherry; EF1-DIO-mCherry), floxed hM3Dq-mCherry (hSyn-DIO-hM3Dq-mCherry) and floxed hM4Di-mCherry (hSyn-DIO-hM4Di-mCherry) AAVs (10^12^ vc/mL; serotype 5) were purchased from the University of North Carolina Vector Core (Chapel Hill, NC).

### Stereotaxic surgeries

Mice were anesthetized with isoflurane and mounted onto a stereotaxic frame. The AAV vectors were infused bilaterally into the CA1 region of the hippocampus (AP: −1.6 mm, DV: −1.7 mm, ML: ±1.1 mm). A volume of 0.5 μL was infused using a thin glass needle connected by Tygon tubing to a 10 μL Hamilton needle syringe by pressure ejection at a rate of 0.1 μL/min. Following infusion, the needle was left in place for 5 min to prevent solution backflow. After surgery, mice were single-housed and experiments were conducted 4 weeks post injection.

### Chemogenetic manipulation of PV interneurons

Mice were intraperitoneally injected with CNO dissolved in 0.9% saline or with 0.9% saline only. For acute treatment, mice received 5 mg/kg (hM4Di) or 2 mg/kg (hM3Dq) CNO 30 min before each training session in the MWM. For prolonged manipulations, mice received 3 mg/kg (hM4Di) CNO daily for 3 weeks. Prolonged treatment was stopped 48 h or 8–10 weeks before behavioral or electrophysiological testing.

### Slice preparation and electrophysiology

After decapitation, brains were quickly removed and stored in ice-cold partial sucrose solution containing (in mM): sucrose 70, NaCl 70, NaHCO_3_ 25, KCl 2.5, NaH_2_PO_4_ 1.25, CaCl_2_ 1, MgSO_4_ 5, sodium ascorbate 1, sodium pyruvate 3 and D(+)-glucose 25 (carboxygenated with 5% CO_2_/95% O_2_). Coronal slices (300 µm thick) from the hippocampus were prepared in ice-cold partial sucrose solution using a microtome (Leica) and incubated for 30 min in 34 °C holding aCSF containing (in mM): NaCl 125, NaHCO_3_ 25, KCl 3, NaH_2_PO_4_ 1.2, CaCl_2_ 1.3, MgSO_4_ 1, MgCl_2_ 1, sodium pyruvate 3, sodium ascorbate 1 and D(+)-glucose 25 (carboxygenated with 5% CO_2_/95% O_2_). After preparation, slices were maintained for at least 1 h in holding ACSF at room temperature. PV cells were identified by mCherry fluorescence. The slicing was done with dimmed lights and the slices were placed in a dark-slicing chamber in order to prevent exposing fluorescently labeled cells to excessive light. Slices were then transferred to the recording chamber and continuously perfused with standard ACSF at 34 °C containing (in mM): NaCl 125, NaHCO_3_ 25, KCl 3, NaH_2_PO_4_ 1.2, CaCl_2_ 1.3, MgSO_4_ 1 and D(+)-glucose 25 (carboxygenated with 5% CO_2_/95% O_2_).

Pyramidal and PV cells in CA1 were recorded in whole-cell mode using a Multiclamp 700B amplifier (Molecular Devices, Sunnyvale, CA). Borosilicate glass electrodes (Harvard Apparatus, Holliston, MA) with tip resistances of 2–5 MOhm were filled with intracellular solution containing (in mM) for pyramidal cells sIPSC and sEPSC recordings: Cs-gluconate 120, CsCl 10, HEPES 10, K-phosphocreatine 10, ATP-Mg 2, and GTP 0.3 EGTA 0.2 QX314 1 (pH adjusted to 7.2 with CsOH) and for all other PV and pyramidal cells recordings: K-gluconate 105, HEPES 7, 10, K2-phosphocreatine 10, ATP-Mg 4, KCl 10, NaCl 20, EGTA 0.2 and GTP 0.3 (pH adjusted to 7.2 with KOH). Resting membrane potential was recorded directly after obtaining whole-cell configuration. Passive and active membrane properties of PV and pyramidal neurons were measured in current-clamp mode by injecting 1 ms of increasing current stimuli. All cells were kept at −65 mV. Access resistance was monitored during the recordings and neurons with access resistance that was above 25 MOhm were excluded from the analysis. Average access resistance for spontaneous postsynaptic currents is displayed in Supplementary Table 10. Neurons that displayed unstable resting membrane potential or aberrant spiking pattern were excluded from the analysis. Data analysis was conducted using a custom-designed script in Igor Pro-6.0 (Wavemetrics). Input resistance was calculated from hyperpolarizing current injections. The data was not corrected for liquid junction potential. IPSCs were recorded at 0 mV holding potential. EPSCs were recorded at −70 mV holding potential. Synaptic events were detected using Mini Analysis Program (Synaptosoft, Decatur, GA). Resting membrane potentials were recorded in voltage-clamp mode in normal aCSF for a minimum of 5 min before the addition of 50 μM CNO for 10 min.

### Preparation and application of soluble Aβ peptides

Aβ peptides were prepared as described previously [24]. Aβ_1–40_, Aβ_1–42_, and reverse peptides were purchased from Bachem (Torrance, CA), dissolved in DMSO and aliquoted before freezing at −20 °C. On the day of the experiment, Aβ peptides were diluted with ACSF at a concentration of 1 μM using a 1:1 mixture of Aβ_1–40_ and Aβ_1–42_. Hippocampal slices were incubated with this solution for 1 h before being transferred to standard aCSF for electrophysiological recording. Control slices were exposed to the reverse peptides.

### BACE1 inhibition

NB-360 was used to inhibit Aβ production in vivo as described previously [39, 40]. In brief, 8–10-week-old APP/PS1-PV-Cre mice expressing mCherry in PV neurons received NB-360 incorporated in food pellets (0.25 g/kg; Provini Kliba, Switzerland; daily oral dose of 20 μmol/kg) or vehicle control food for 6–8 weeks. Following the treatment (at 15–17 weeks of age), patch-clamp recordings were performed to measure excitability of hippocampal CA1 PV interneurons and ELISA was performed to measure hippocampal Aβ levels.

### Tissue preparation and immunohistochemistry

Staining was performed on free-floating brain sections as previously described [37]. Sections were blocked with 0.2% (v/v) Triton X-100 and 5% (v/v) fetal bovine serum in PBS, and incubated overnight with mouse anti-PV (Millipore, Billerica, MA, MAB 1572; 1:1000) for PV staining or 6E10 (Signet, Dedham, MA; 1:800) for Aβ staining. PV and Aβ staining were visualized using anti-mouse Alex568-labeled secondary antibodies (Invitrogen, Carlsbad, CA; 1:400), incubated for 2 h at RT. Sections were washed and coverslipped in Vectashield including DAPI as a nuclear dye (Vector Laboratories, Burlingame, CA). PV staining was quantified using ImageJ v1.48. PV- and mCherrry-positive cells were counted using image thresholding and automated particle analysis in ImageJ (v1.48). All images were acquired on a Leica DM5000 fluorescence microscope using three different filter cubes (L5, TX2, and Y5) and with a DFC360FX camera (12 bits resolution). For each experiment, identical objective, exposure time, gain settings, and camera settings were used for all images.

### ELISA

Frozen mouse hippocampi were solubilized by sonication in 0.4% diethylamine (DEA) in 5 M NaCl. After centrifugation at 135,000 × *g* for 1 h at 4 °C, the supernatant containing the soluble Aβ fraction was recovered, neutralized by adding a 1/10 volume of 0.5 M Tris-HCl, pH 6.8. ELISA was performed using the V-PLEX Aβ Peptide Panel 1 (6E10) Kit (Mesoscale Discovery, Rockville, MD) according to manufacturer’s instruction.

### Behavioral assays

Spatial memory was tested in a MWM setup. Before testing, mice were handled for 5 days. A circular pool (ø 1.2 m) was filled with water which was made nontransparent with nontoxic white paint and kept at a temperature of 23 ± 2 °C. An escape platform (ø 9 cm) was placed at 30 cm from the edge of the pool submerged 0.5 cm below the water surface. Visual cues were located around the pool at a distance of ~1 m. During testing lights were dimmed and covered with white sheets and mice were video-tracked using Viewer (BiobServe, Fort Lee, NJ). Mice were trained for 4 consecutive days using 4 trials/day with a 30–180 s intertrial interval. In each trial, mice were placed in the water at a random start position and allowed a maximum of 60 s to find the platform. Mice that were unable to find the platform within 60 s were placed back on the platform by hand. After 15 s on the platform, mice were tested again. To prevent hypothermia mice were placed in their home cage for 3 min between trials 2 and 3. On day 5 a probe trial was performed with the platform removed. Mice were placed in the pool opposite from the platform location and allowed to swim for 60 s. During training trials, the latency to reach the platform was measured; in the probe trial, the time spent in each quadrant of the pool was measured. Memory was assessed as the amount of time spent in the target quadrant.

### Data collection and statistical analysis

Sample sizes were chosen based on previous experiments and pilot studies. All statistical analyses were performed using a Student’s *t* test for two-group comparison for groups with equal variances, a Wilcoxon–Mann–Whitney rank test for two-group comparison of nonparametric data with unequal variances or two-way ANOVA for three- or four-group comparisons with LSD test or Student’s *t* test for post-hoc analysis. Two-way repeated measures ANOVA was used for assessing effects within groups and between groups in electrophysiological and behavioral experiments with repeated measurements in the same cell or animal. For comparing cumulative frequency distributions, Kolmogorov–Smirnov *Z* test was used. All tests were two-sided. All quantitative data are represented as means ± standard errors of the means. To reduce selection bias, all mice were randomly allocated to the different groups according to their genotypes. Post-hoc analysis of DREADD expression was used to confirm specific targeting of CA1 PV interneurons in mice; Animals with an off-target DREADD expression were excluded from the analysis. Experimenters were blind to the treatment and genotype of mice when analyzing the data.

## Supplementary information

Supplementary Figures 1–10

Supplementary Tables 1–10
