# Supplementary material for: Early restoration of parvalbumin interneuron activity prevents memory loss and network hyperexcitability in a mouse model of Alzheimer’s disease
Source: Mol Psychiatry. 2019 Aug 20;25(12):3380–98. doi: 10.1038/s41380-019-0483-4 (PMC7714697; doi:10.1038/s41380-019-0483-4)
Supplement: Supplementary file 1 — Supplementary Figures 1–10 [file 41380_2019_483_MOESM1_ESM.pdf]

Early restoration of parvalbumin interneuron activity prevents memory loss and network hyperexcitability in a mouse model of Alzheimer's disease

Sara Hijazi, Tim S Heistek, Philip Scheltens, Ulf Neumann, Derya R. Shimshek, Huibert D Mansvelder, August B Smit & Ronald E van Kesteren

SUPPLEMTARY FIGURES 1-10

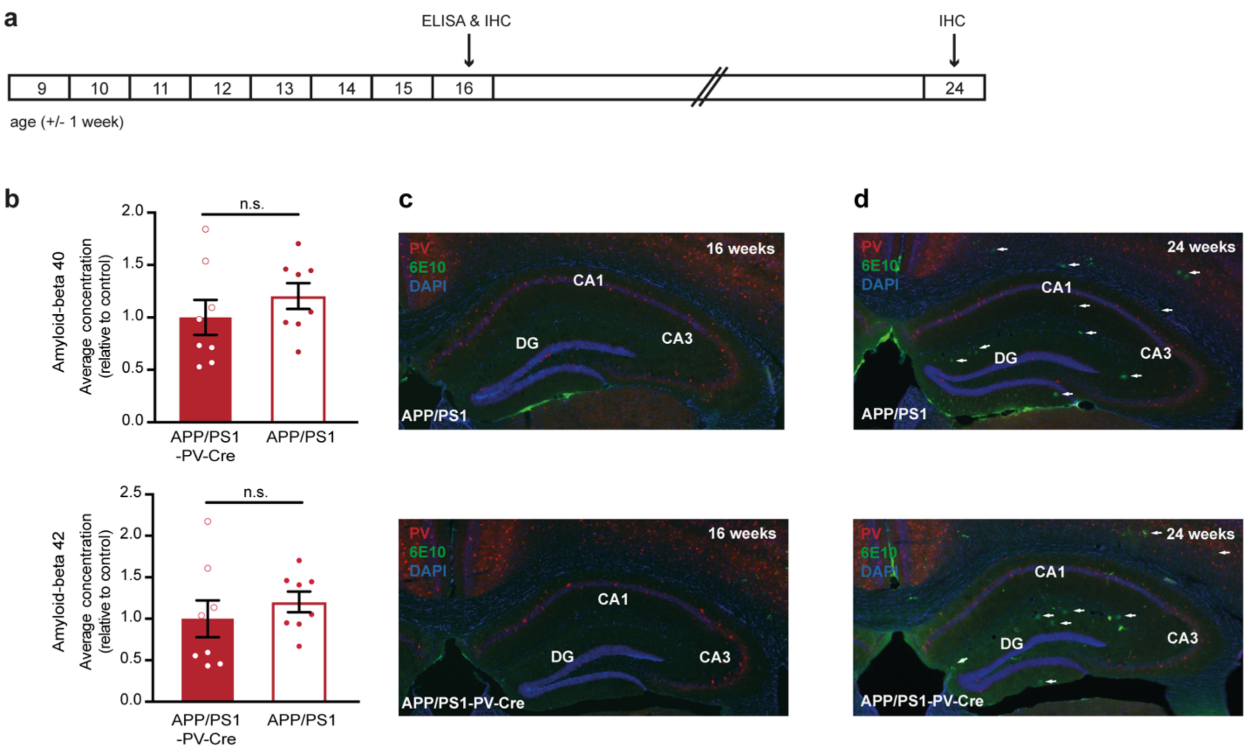

Supplementary Figure 1. Amyloid state in young APP/PS1 and APP/PS1-PVCre mice. (**a**) APP/PS1 and APP/PS1-PV-Cre mice were sacrificed at 16 and 24 weeks of age to measure Aβ levels using enzyme-linked immunosorbent assay (ELISA) and immunohistochemistry (IHC). (**b**) Relative $A\beta_{1\text{–}40}$ (upper panel) and $A\beta_{1\text{–}42}$ (lower panel) levels in the hippocampus of 16 weeks-old APP/PS1-PV-Cre and APP/PS1 mice (Student's t-test; n = 8 mice per group; p = 0.344 and p = 0.550, respectively). (**c**) Representative images of 6E10 (green) and PV immunostaining (red) in the CA1 region of APP/PS1 (upper panel) and APP/PS1-PV-Cre (lower panel) mice at 16 weeks of age indicating absence of amyloid plaques. (**d**) Representative images of 6E10 (green) and PV immunostaining (red) in the CA1 region of APP/PS1 (upper panel) and APP/PS1-PV-Cre (lower panel) mice at 24 weeks of age showing aggregation of amyloid plaques (indicated with white arrows).

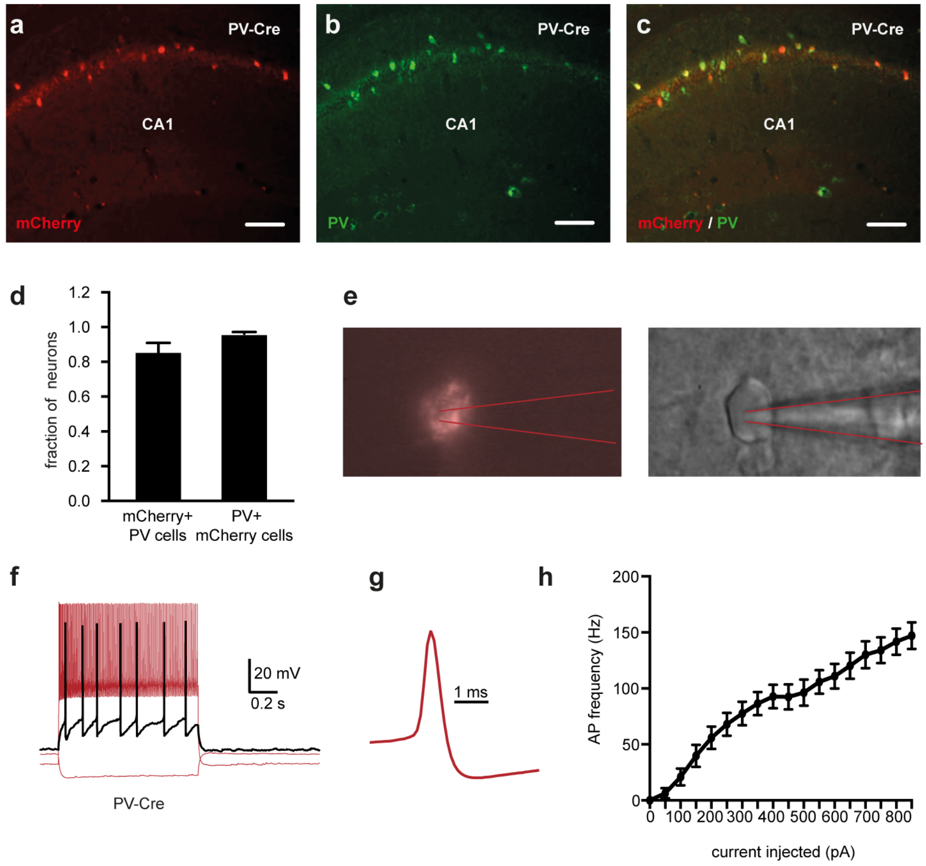

Supplementary Figure 2. Correct targeting of PV-expressing interneurons in the CA1 region of the hippocampus of PV-Cre mice. Representative images of (**a**) mCherry expression (red) and (**b**) PV immunostaining (green) in the CA1 region of PV-Cre mice. (**c**) Overlay of mCherry and PV staining indicating that mCherry expression is restricted to PV neurons. Scale bars: 100 µm. (**d**) Bar graphs showing the percentage of PV-positive cells expressing mCherry and of the percentage of mCherry-positive cells expressing PV (n = 20 sections from 4 mice). (**e**) mCherry-labeled PV interneuron in the CA1 region of the hippocampus (left panel) and live contrast image of the same neuron with the patch pipette attached (right panel). (**f**) Voltage responses to 1 s hyperpolarizing or depolarizing current steps from a PV-expressing interneuron in a WT-PV-Cre mouse. (**g**) Average action potential (AP) waveforms of the first AP from a representative cell with a characteristic narrow AP half-width. (**h**) Average AP frequency in response to 0-850 pA depolarizing current steps illustrating the fast-spiking profile of PV-expressing interneurons (n = 9 cells from 3 mice).

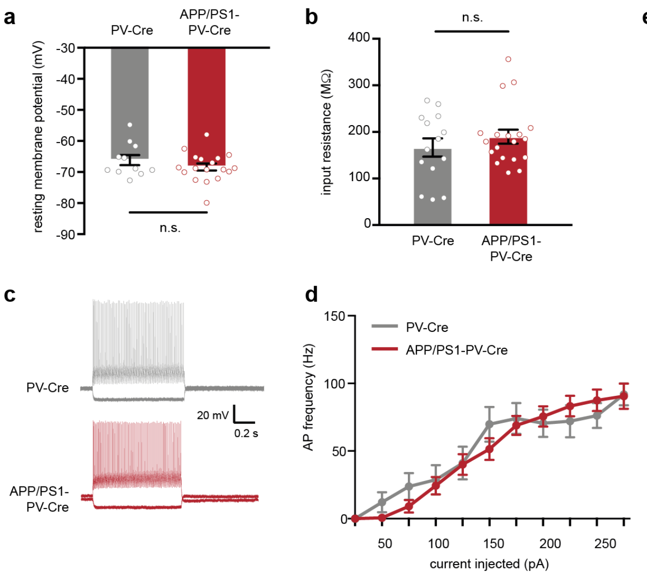

Supplementary Figure 3. Membrane and action potential firing properties of hippocampal PV interneurons and pyramidal neurons are unaffected in APP/PS1 mice at 7 to 9 weeks of age. (**a**) PV neuron resting membrane potential and (**b**) input resistance were unaffected in APP/PS1-PV-Cre mice at 7 to 9 weeks of age compared with PV-Cre wildtype controls (Student's t-test: *n* = 13/17 cells from 4 mice per group; *p* = 0.329 and *p* = 0.344). (**c**) Voltage responses to 1 s hyperpolarizing or depolarizing current steps from a PV neuron in a PV-Cre wildtype mouse (grey) or an APP/PS1-PV-Cre mouse (red). (**d**) Average action potential (AP) frequency in response to 0-250 pA depolarizing current steps showing no changes in PV neuron excitability in APP/PS1-PV-Cre mice compared with PV-Cre wildtype controls (*genotype x current* two-way repeated measures ANOVA: *n* = 13/17 cells from 4 mice per group, $F_{5,140}$ = 0.85, *p* = 0.514). (**e**) Pyramidal neuron resting membrane potential and (**f**) input resistance were unaffected in APP/PS1-PV-Cre mice at 7-9 weeks of age compared with PV-Cre wildtype controls (Student's t-test: *n* = 10/12 cells from 3 mice per group; *p* = 0.755 and *p* = 0.935). (**g**) Voltage responses to 1 s hyperpolarizing or depolarizing current steps from a pyramidal neuron in a PV-Cre wildtype mouse (grey) or an APP/PS1-PV-Cre mouse (red). (**h**) Average action potential (AP) frequency in response to 0-250 pA depolarizing current steps showing no changes in pyramidal neuron excitability in APP/PS1-PV-Cre mice compared with PV-Cre wildtype controls (*genotype x current* two-way repeated measures ANOVA: *n* = 10/12 cells from 3 mice per group, $F_{10,110}$ = 0.60, *p* = 0.811).

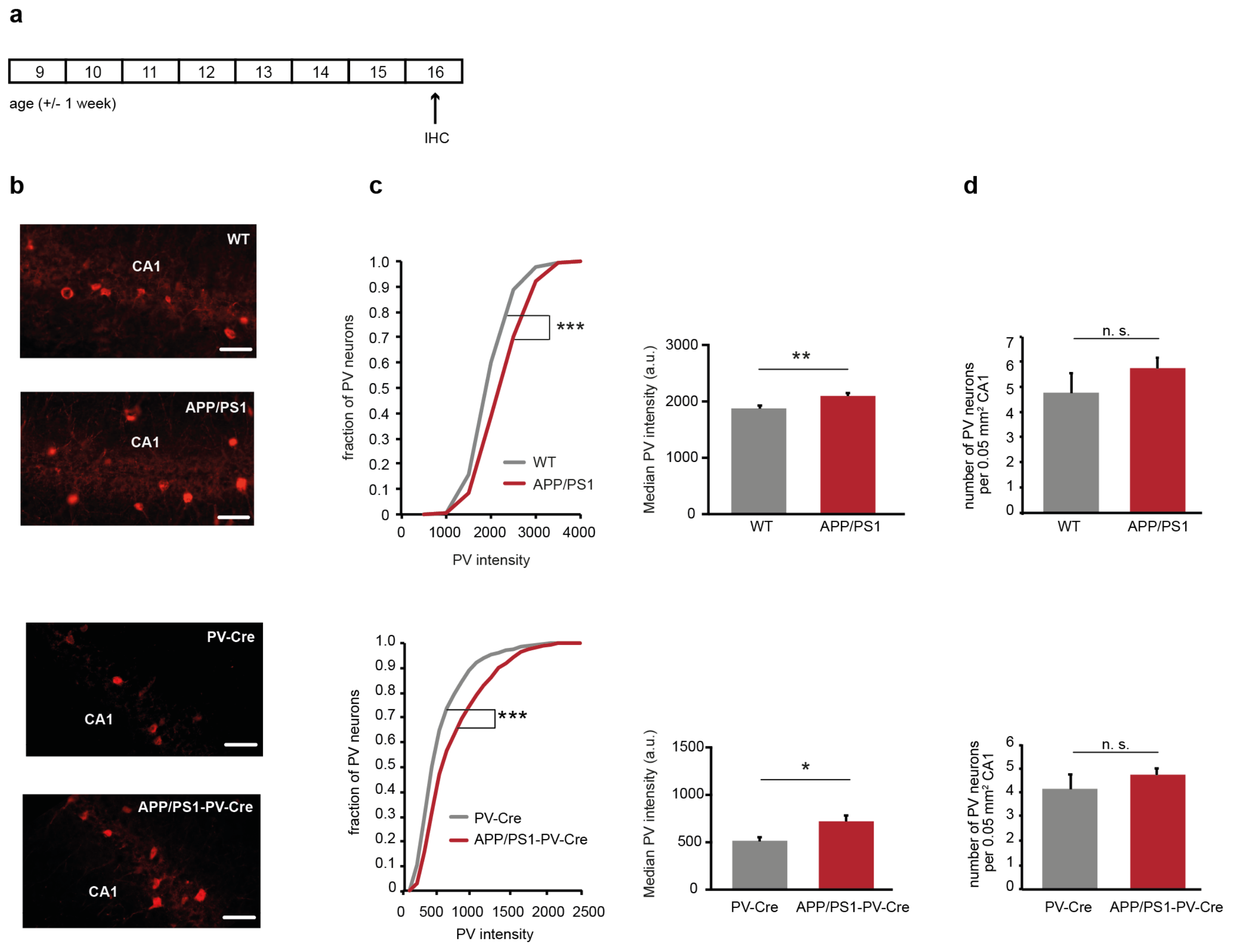

Supplementary Figure 4. PV interneurons show increased PV immunoreactivity in APP/PS1 mice at 15-17 weeks of age. (**a**) APP/PS1 and APP/PS1-PVCre mice were perfused at 15-17 weeks of age for immunohistochemical (IHC) analysis. (**b**) PV neurons were visualized in coronal sections of the hippocampus of APP/PS1 (upper panel) and APP/PS1-PV-Cre (lower panel) mice and their respective wildtype (WT) and PV-Cre controls at 15-17 weeks of age using anti-PV staining (red). PV intensities per neuron were quantified in high magnification images of the CA1 area only. CA, cornus ammonis. Scale bars: 40 µm. (**c**) Cumulative frequency distribution of PV intensities of all cells measured shows an overall increase in PV intensities in APP/PS1 (upper panel) and APP/PS1-PV-Cre (lower panel) mice compared with WT and PV-Cre controls (Kolmogorov-Smirnov Z test: n = 1042/1130 cells from 16 sections from 4 mice per genotype for APP/PS1 vs WT; n = 1516/1558 cells from 16 sections from 4 mice per genotype for APP/PS1-PV-Cre vs WT-PV-Cre , ***$p < 0.001$). (**d**) Median PV intensity of all cells measured shows a significant increase in APP/PS1 (upper panel) and APP/PS1-PV-Cre (lower panel) mice compared with WT and PV-Cre controls (Mann-Whitney test: n = 16 sections from 4 mice per genotype, *$p < 0.05$ and **$p < 0.01$). (**e**) Quantification of PV-positive cells in the CA1 area confirmed that the total number of PV-positive cells is unaltered between genotypes (Student's t test: n = 4 mice per genotype for APP/PS1 vs WT, p = 0.420; n = 4 mice per genotype for APP/PS1-Pv-Cre vs PV-Cre, p = 0.275).

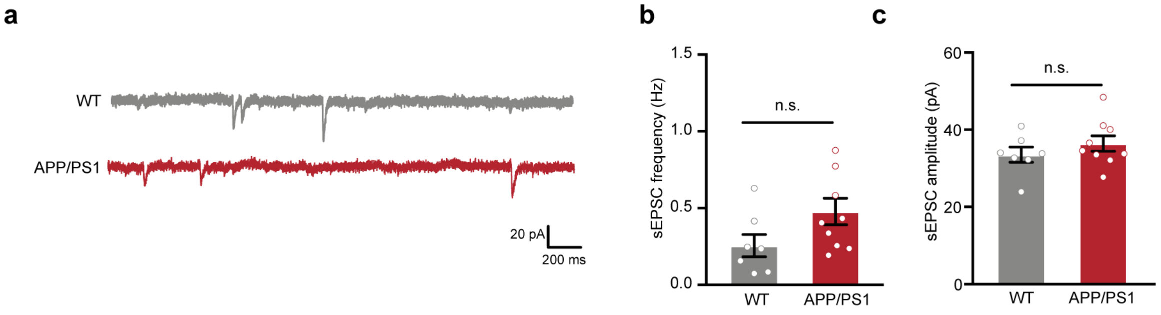

Supplementary Figure 5. Excitatory transmission onto pyramidal neurons is unaltered in APP/PS1 mice at 15-17 weeks of age. (**a**) Example traces of spontaneous excitatory postsynaptic currents (sEPSC) recorded from hippocampal pyramidal neurons in APP/PS1 mice (red) and WT controls (grey) at 15-17 weeks of age. (**a-c)** No significant alterations were observed in the frequency (**b**) or in the amplitude (**c**) of sEPSCs in APP/PS1 mice compared with WT controls (Mann-Whitney test: n = 7/9 cells from 3 mice per genotype, p = 0.12).

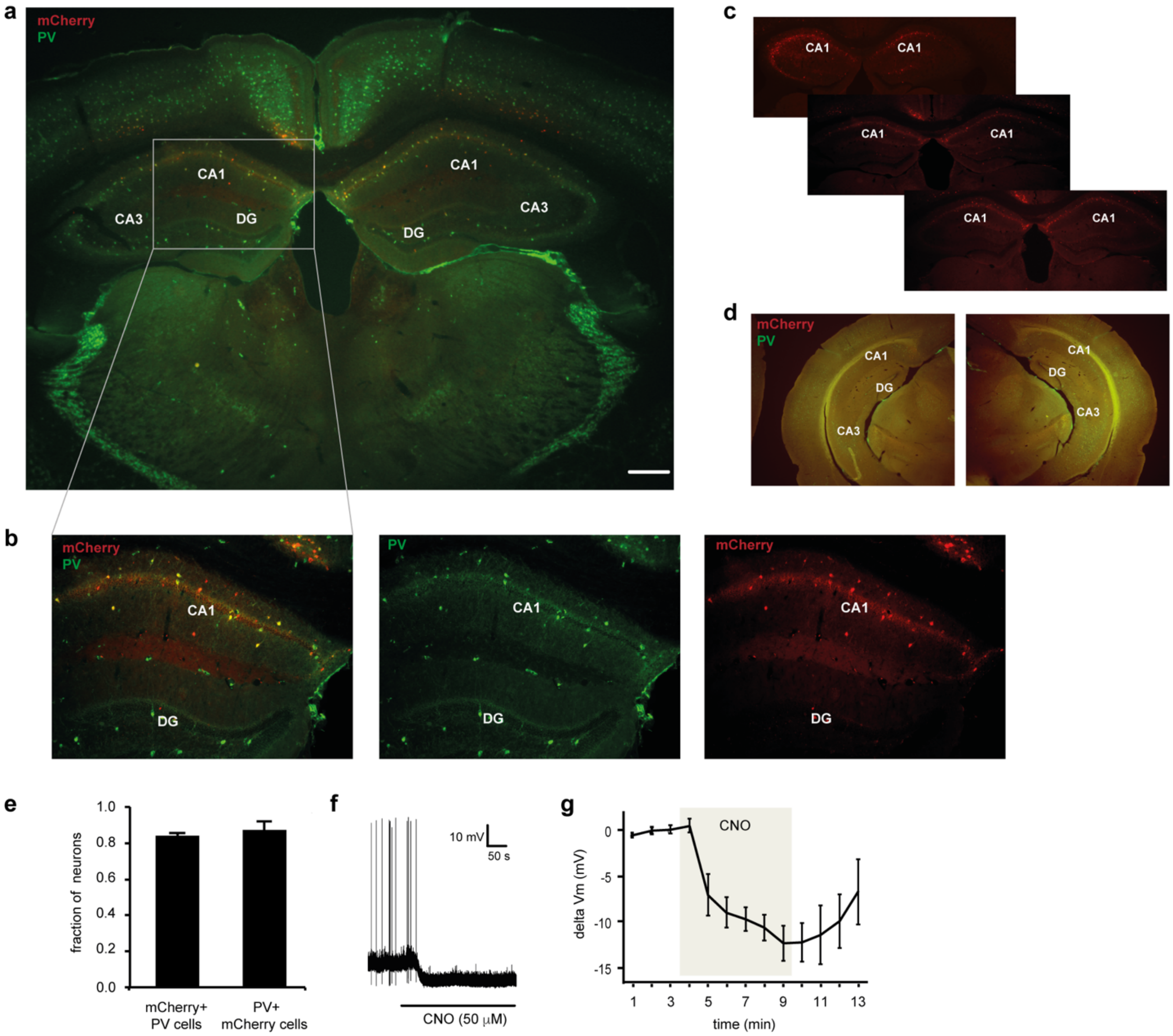

Supplementary Figure 6. Characterization of DREADD expression and function in the brain. (**a**) Representative overview composite image of mCherry expression (red) and PV immunostaining (green) in a coronal slice of a PV-Cre mouse; scale bar: 200 μm. (**b**) Detailed images of the CA1 region of the hippocampus in higher magnification; scale bars: 75 μm. (**c**) Sections covering the dorsal hippocampus, from -1.0 mm to -3.0 mm relative to bregma, showing that DREADD expression is restricted to the CA1 region. (**d**) Sections covering the ventral hippocampus showing absence of DREADD expression in this region. (**e**) Bar graphs showing the percentage of PV-positive cells expressing the hM4Di receptor and of the percentage of hM4Di-mCherry-positive cells expressing PV (*n* = 18 sections from 4 mice). (**f**) Representative voltage trace of 50 μM CNO-induced hyperpolarization in an hM4Di-positive neuron. (**g**) Average membrane potential of hM4Di-positive cells before and after CNO application (*n* = 7 cells from 2 mice).

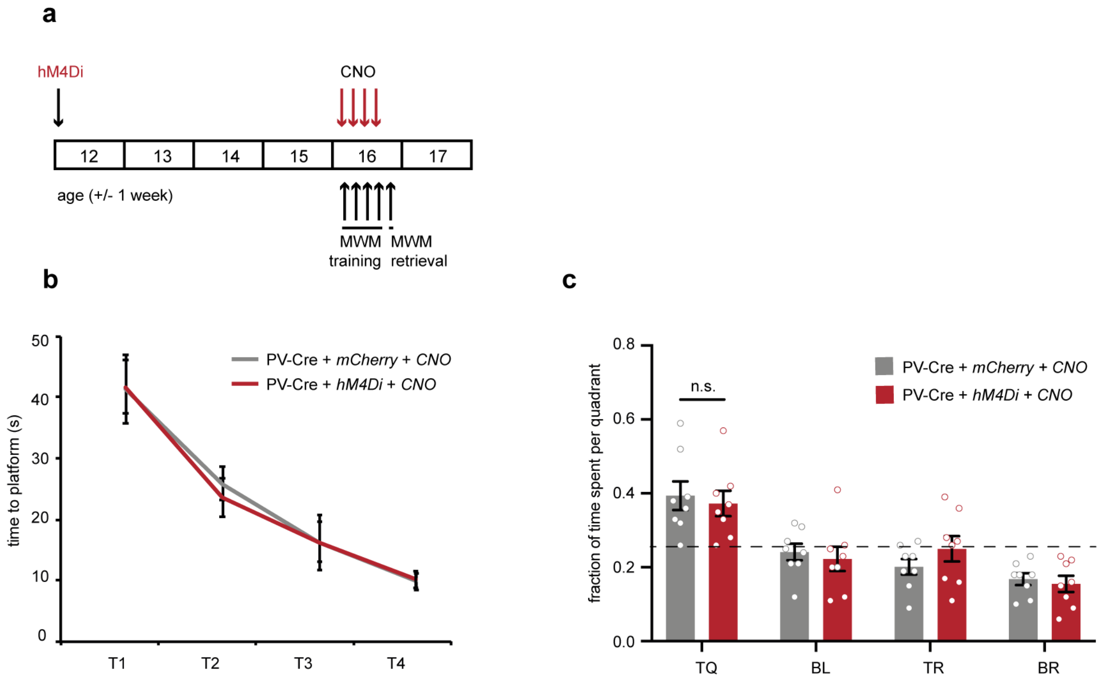

Supplementary Figure 7. Acute chemogenetic inhibition of hippocampal PV neurons does not affect spatial memory in wildtype mice). (**a**) WT-PV-Cre mice were injected with hM4Di virus at 11-13 weeks of age and tested in a MWM test four weeks later. CNO (5 mg/kg) or saline were i.p. injected 30 min prior to each training session. (**b**) WT-PV-Cre mice that expressed hM4Di and had received CNO injections showed no difference in learning compared with mCherry-expressing PV-Cre mice (*group x training* two-way repeated measures ANOVA: $n = 8$ mice per group, $F_{3,21} = 0.22$, $p = 0.880$). (**c**) During the one-minute probe trial, CNO-treated WT-PV-Cre mice expressing hM4Di spent an equal amount of time in the target quadrant (TQ) compared with WT-PV-Cre mice expressing mCherry (two-way ANOVA: $n = 8$ mice per group, $F_{3,21} = 4.33$, $p = 0.731$). Compared with chance level (dashed line), both hM4Di- and mCherry-expressing mice spent significantly more time in the target quadrant, confirming that the absence of memory impairments in both groups (Student's t-test; $p < 0.01$).

a

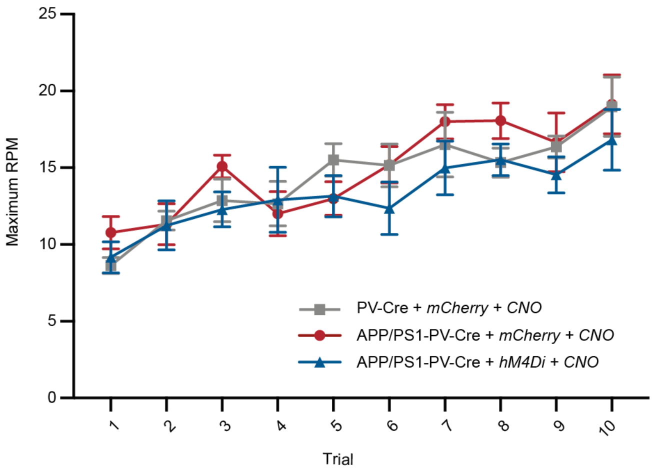

Supplementary Figure 8. Chemogenetic inhibition of hippocampal PV neurons does not affect locomotor behavior. (**a**) CNO-treated APP/PS1-PV-Cre mice expressing hM4Di, WT-PV-Cre mice expressing mCherry and APP/PS1-PV-Cre mice expressing mCherry show no differences in motor skills in a rotarod test (*group x trial* two-way repeated measures ANOVA: n = 7 mice per group type, $F_{2,18} = 0.8631$, $p = 0.439$).

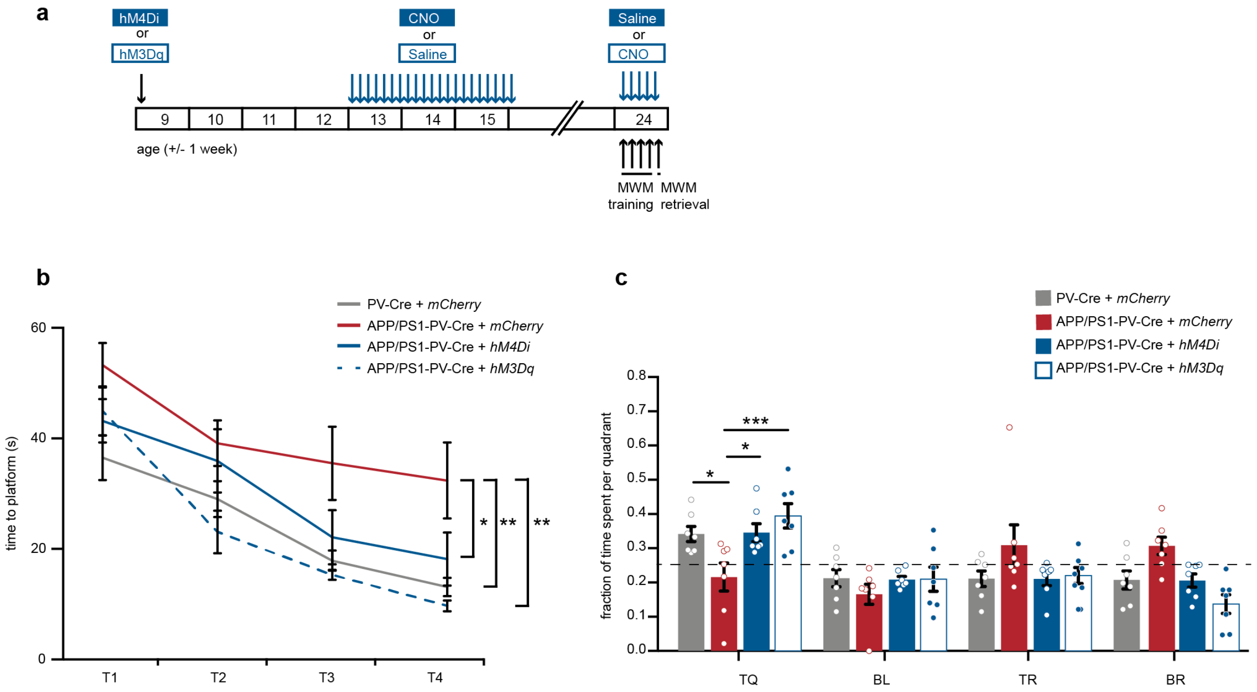

Supplementary Figure 9. Chemogenetic activation of hippocampal PV neurons rescues spatial memory deficits in APP/PS1 mice at 23-25 weeks of age, similar to chemogenetic inhibition at 13-15 weeks of age. (**a**) Animals were injected with either hM4Di or hM3Dq virus at 8-10 weeks of age. After 4 weeks, CNO (3 mg/kg) was i.p. injected daily for a period of 3 weeks in hM4Di-expressing APP/PS1-PVCre, mCherry-expressing WT-PV-Cre and mCherry-expressing APP/PS1-PV-Cre mice; saline was i.p. injected daily for a period of 3 weeks in hM3Dq-expressing APP/PS1-PVCre mice. At weeks 23-25, 30 minutes prior to each MWM session, hM3Dq-expressing APP/PS1-PVCre mice received i.p. injections of CNO (1 mg/kg); all other groups received saline injections. (**b**) Spatial learning was assessed measuring the time required to find the hidden platform on 4 consecutive training days (T1-4). APP/PS1-PV-Cre mice that expressed hM3Dq and received CNO injections showed significantly improved learning compared with mCherry-expressing APP/PS1-PV-Cre mice that received saline, and were comparable to both hM4Di-expressing APP/PS1-PVCre mice that received CNO injections in week 13-15 and WT-PV-Cre mice that received saline injections (*group x training* two-way repeated measures ANOVA post-hoc LSD test: n = 7/7/8/7 mice per group, $F_{3,25} = 4.79$, $p = 0.009$; post-hoc LSD test: *$p < 0.05$, **$p < 0.01$). (**c**) During the one-minute probe trial, CNO-treated APP/PS1-PV-Cre mice expressing hM3Dq spent significantly more time in the target quadrant (TQ) compared with saline-treated APP/PS1-PV-Cre mice expressing mCherry, and performed similar to earlier treated hM4Di-expressing APP/PS1-PVCre mice and saline-treated WT-PV-Cre mice (two-way ANOVA: n = 7/7/8/7 mice per group, $F_{9,54} = 3.56$, $p = 0.001$; post-hoc LSD test: *$p < 0.05$, ***$p < 0.001$). Compared with chance level (dashed line), CNO-treated APP/PS1-PV-Cre expressing hM3Dq, saline-treated APP/PS1-PV-Cre expressing hM4Di and saline-treated WT-PV-Cre mice spent significantly more time in the target quadrant, while control APP/PS1-PV-Cre control mice did not (Student's t test: $p < 0.05$ for WT-PV-Cre and hM4Di-expressing APP/PS1-PV-Cre mice; $p < 0.01$ for hM3Dq-expressing APP/PS1-PV-Cre).

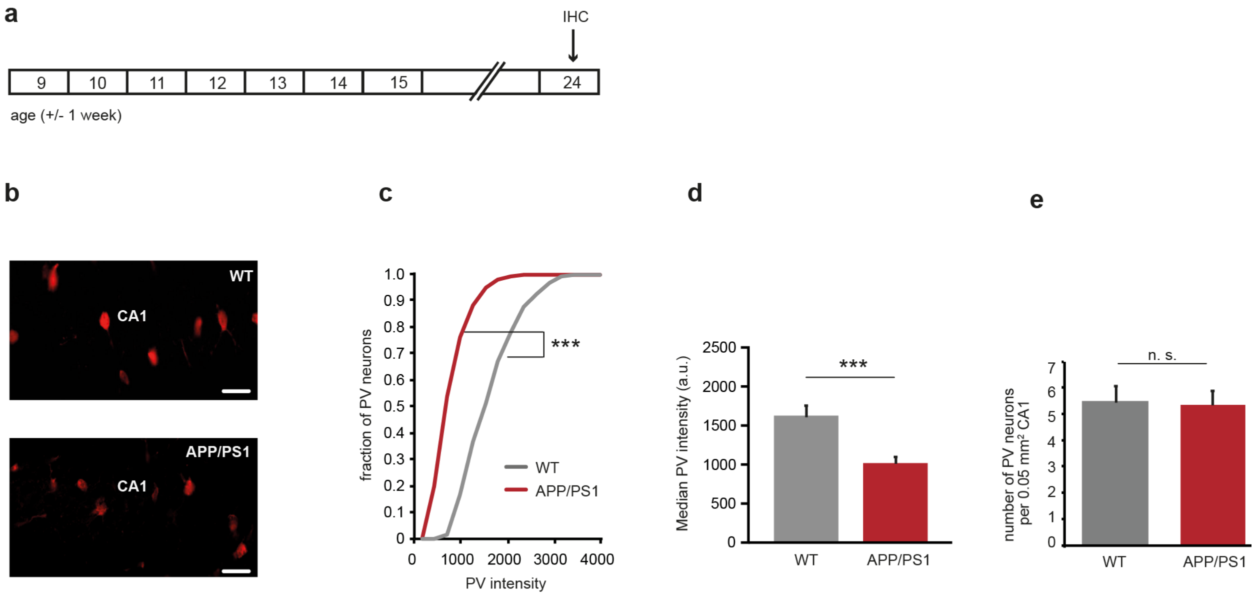

Supplementary Figure 10. PV interneurons show decreased PV immunoreactivity in APP/PS1 mice at 23-25 weeks of age. (**a**) APP/PS1 mice were perfused at 23-25 weeks of age for immunohistochemical (IHC) analysis. (**b**) PV neurons were visualized in coronal sections of the hippocampus of APP/PS1 mice and wildtype (WT) controls using anti-PV staining (red). PV intensities per neuron were quantified in high magnification images of the CA1 area only. Scale bars: 40 µm. (**c**) Cumulative frequency distribution of PV intensities of all cells measured shows an overall decrease in PV intensities in APP/PS1 mice compared with WT controls (Kolmogorov-Smirnov Z test: n = 1065/1027 cells from 16 sections from 4 mice per genotype, ***$p < 0.001$). (**d**) Median PV intensity of all cells measured shows a significant decrease in APP/PS1 compared with WT controls (Mann-Whitney test: n = 15/16 sections from 4 mice per genotype, ***$p < 0.01$). Quantification of PV-positive cells in the CA1 area confirmed that the total number of PV-positive cells is unaltered between genotypes (Student's t test: n = 4 mice per genotype for APP/PS1 vs WT, $p = 0.318$).
